# Nurses’ perceptions of leadership in Brazilian hospitals during COVID-19 through Kotter’s conception*

**DOI:** 10.1590/1518-8345.7151.4281

**Published:** 2024-08-19

**Authors:** Patrícia Alves Galhardo Varanda, Gilberto Tadeu Reis da Silva, Simone Coelho Amestoy, Vânia Marli Schubert Backes, Gabriela Marcellino de Melo Lanzoni, Augusto Ferreira Umpiérrez, Naomy Safira Batista da Silva

**Affiliations:** 1 Universidade Federal da Bahia, Escola de Enfermagem, Salvador, BA, Brazil.; 2 Scholarship holder at the Coordenação de Aperfeiçoamento de Pessoal de Nível Superior (CAPES), Brazil.; 3 Universidade Federal do Vale do São Francisco, Departamento de Enfermagem, Petrolina, PE, Brazil.; 4 Universidade Federal de Santa Catarina, Departamento de Enfermagem, Florianópolis, SC, Brazil.; 5 Universidad Católica del Uruguay, Departamento de Enfermagem, Montevideo, Montevideo, Uruguai.

**Keywords:** Nursing, Leadership, Nurses, COVID-19, Change Management, Pandemics

## Abstract

**Objective:**

to understand nurses’ leadership perceptions during the COVID-19 pandemic in Brazilian university hospitals, through the lens of John Kotter’s concepts and his eight-step change model.

**Method:**

a multicenter qualitative research with an analytical design. The sample comprised 139 nurses working in COVID-19 settings, interviewed using a semi-structured interview guide. Data were categorized through Bardin’s content analysis based on John Kotter’s concepts and his eight-step change model.

**Results:**

the findings yielded significant insights into nurses’ perceptions of leadership during the COVID-19 response, which exhibited characteristics consistent with Kotter’s principles. There is evidence of leadership based on the pursuit of knowledge, grounded in polished communication, facilitating teamwork through a relationship of trust and respect. The recognition of the complexity and difficulty of exercising nursing leadership, particularly in crisis contexts, is apparent.

**Conclusion:**

nurses’ perceptions reinforce essential elements for leadership practice, such as the importance of seeking diverse knowledge, polished communication, relationships based on trust and respect, and recognition of the complexity of leadership, thus presenting characteristics in line with Kotter’s principles.

## Highlights

**(1)** Nurses’ perceptions of leadership during COVID-19 were highlighted.

**(2)** COVID-19 intensified the complexity in nurses’ leadership roles.

**(3)** Nurses’ leadership was consolidated in knowledge, communication, trust.

**(4)** Working in the COVID-19 response as a nurse-leader has become challenging.

**(5)** Kotter’s principles assist in reflections on nurses’ leadership.

## Introduction

Leadership is indispensable to nursing practice, and it is important for nurses to seek its development. Although definitions of leadership may be complex, we recognize great leadership when we see it^1^ ^)-(^ ^2^. In nursing, leadership can be characterized as a fundamental professional competence in both clinical and administrative practice, where nurses guide, support, and direct their health care teams, enabling positive outcomes in patient care^3^ ^)-(^ ^4^.

Nurses constitute the largest global health care workforce, and in some situations, they are the sole available providers^2^. Thus, they play crucial roles in health care practice, education, and research. The World Health Organization (WHO)^5^ ^)-(^ ^6^ emphasizes strengthening nurses’ leadership to influence health policies and decisions. Therefore, in both normal times and during pandemics, nurses play a vital frontline role in health care, significantly contributing to disease management^5^ ^)-(^ ^6^. 

COVID-19 began in 2019 in Wuhan, China, causing symptoms ranging from a simple cold to acute respiratory syndrome (SARS-CoV-2), known as Coronavirus Disease 2019 (COVID-19)^7^. Quickly, it became one of the worst health catastrophes, impacting health care systems worldwide^8^. In March 2020, the WHO declared COVID-19 a pandemic. On May 5, 2023, the WHO announced that COVID-19 was no longer a public health emergency due to reductions in hospitalization rates, ICU admissions, and global mortality^9^ ^)-(^ ^10^.

However, the pandemic consequences are concerning and can be observed across various sectors. Among these, hospitals were abruptly challenged to expand intensive care unit beds, provide training for health care professionals to care for COVID-19 patients, and acquire a larger quantity of personal protective equipment. They were dealing with an unknown virus with high contagion and high lethality. Brazilian federal university hospitals (HUFs) were designated as references for medium and high complexity care within the Unified Health System (SUS) for COVID-19 patient assistance^9^ ^),(^ ^11^.

Within management, nurse leaders formed committees aimed at planning and quickly organizing service provision flows (care pathways), making adjustments to ICU infrastructure, and developing protocols related to the care of patients hospitalized due to COVID-19. Another noteworthy aspect of these professionals’ performance during the pandemic was their coordination among the multiprofessional team to make necessary adjustments to promote holistic care, with standardized operational procedures, safety checklists, and updating of clinical practice guidelines whenever available^12^.

This pandemic, therefore, caused a global crisis and, in the context of nursing, provided an opportunity to highlight the nurse’s leadership in facing it^13^. A study^14^ noted that nurses demonstrated a strong sense of responsibility and pride in providing nursing care to patients with COVID-19. Nurse managers and specialists played crucial roles in coordination during the pandemic, and nursing leadership stood out as a crucial element in maintaining this, including maintaining a good relationship between nursing and patients^14^.

In this context of extreme uncertainties, the dedication of nursing assumed immeasurable value. The leadership skills of nurses globally were put to the test, their actions proving essential for future generations^15^, highlighting the need for these professionals to be prepared to handle such situations, which may occur again, even if for different reasons^15^. Thus, studies that highlight elements capable of contributing to the development of nursing leadership are important and pressing, especially in crisis scenarios, where this competence proves to be fundamental.

In this sense, the present study was proposed, which can further strengthen the contributions of this type of research by focusing on the concepts of John Kotter’s theoretical framework and his eight-step change model. The author, internationally recognized for his study of leadership and change in organizations, asserts that leadership is a set of intrinsic processes of adaptable organizations to significantly modify circumstances, aiming for the future, aligning and motivating people^16^. 

Kotter is the creator of the “eight-step change process” model, conceptualized in his work “Leading Change.” The adoption of such a model allows the leader to avoid the eight most common errors in a change process and effectively implement it. The model consists of countering these eight errors identified during the following stages: Sense of Urgency (reasons for change); Administrative Coalition (agents of change); Vision and Strategy (clear and objective forward thinking); Communication (properly communicated); Empowerment (empowering people); Short-term Wins (motivating progress); Consolidating Gains (maintaining focus); and New Methods in Culture (incorporating change)^16^ ^)-(^ ^18^.

Kotter’s eight-step model is an empowering tool for leaders to effectively implement changes, which can be applied in the health care context. Therefore, it’s important for nursing leaders to seek understanding and application of the model regarding change processes and contribute to improvements in the quality of care. These are interconnected and complementary steps to ensure effective change, thus, depending on the scenario, some steps may occur simultaneously. Therefore, the author emphasizes the importance of not skipping any stage of the change process, and when all stages are well conducted, successful results are achieved. However, in the complex world we live in, some cases may not strictly follow the flow of eight steps, but the pattern of the eight stages is the basic paradigm observed in successful change processes^16^ ^)-(^ ^18^.

During this time, international studies^19^ ^)-(^ ^21^ conducted in the Philadelphia Metropolitan Area, Africa, and the United Kingdom applied John Kotter’s model in the health care context through nursing leaders and other health care professionals during the COVID-19 pandemic. Positive results emerged from Kotter’s change model and its leadership concepts, although in some cases, professionals needed to change their beliefs, values, behaviors, and attitudes in order to continuously develop new understandings, thus enabling the successful change adoption by the majority of professionals^19^ ^)-(^ ^21^. However, no studies were found in the context of nursing in Latin America. 

Given this, the following question arose: what are the perceptions of nursing leadership during the COVID-19 pandemic in Brazilian university hospitals, under the concepts of John Kotter? Therefore, to answer this question, the following objective was presented: to understand the perceptions of nursing leadership during the COVID-19 pandemic in Brazilian university hospitals, based on the concepts of John Kotter’s framework and his eight-step change model.

## Method

### Study design

This is a multicenter qualitative study with an analytical design, supported by the theoretical constructs of John Paul Kotter and his eight-step change model. A qualitative design was considered the most suitable to understand and identify nurses’ leadership perceptions in the context of the pandemic. To ensure methodological rigor, the study used items from the checklist for qualitative research according to the Consolidated Criteria for Reporting Qualitative Research (COREQ) guidelines.

### Study location

The study was conducted in ten Brazilian university hospitals, which serve as practical fields for various health-related courses, including nursing. These hospitals handle cases of medium and high complexity for the Unified Health System (SUS)^23^, and eight of them are affiliated with the Brazilian Hospital Services Company (EBSERH). These hospitals are located in the South region (two hospitals, including the main institution); Southeast (two); Northeast (two hospitals, one of which is the proposing institution of the researcher); North (two); and Midwest (two). The selection of these ten university hospitals was intentional, initially through interaction among research groups, faculty, and researchers associated with the educational institutions integrated with these university hospitals, as well as for representing the five regions of Brazil and for being references in the care of COVID-19 patients through SUS during the pandemic. Thus, the first contact with the university hospitals was made through the faculty members who were leading the multicenter project at each of the educational institutions.

The study was conducted in ten Brazilian university hospitals, which serve as practical fields for various health-related courses, including nursing. These hospitals handle cases of medium and high complexity for the Unified Health System (SUS)^23^, and eight of them are affiliated with the Brazilian Hospital Services Company (EBSERH).

These hospitals are located in the following regions: South (two hospitals, including the main institution); Southeast (two); Northeast (two hospitals, one of which is the proposing institution of the researcher); North (two); and Midwest (two). 

The selection of these ten university hospitals was intentional, primarily through interaction among research groups, faculty, and researchers associated with the educational institutions integrated with these university hospitals, as well as for representing the five regions of Brazil. Furthermore, these hospitals are references in the care of COVID-19 patients through SUS during the pandemic. Thus, the first contact with the university hospitals was made through the faculty members who were leading the multicenter project at each of the educational institutions.

### Period

Data collection took place between March 2021 and April 2022.

### Population, selection criteria, sample definition

The sample consisted of a total of 139 participants, and the research participants were nurses providing care to COVID-19 patients without any employment bond. Eligibility criteria included: being a practicing nurse with COVID-19 patients for a minimum of three months, a time period believed to allow for sufficient experience regarding the addressed phenomenon. 

It is worth noting that since this study is linked to a larger multicenter project that used mixed methods, initially, the larger project conducted the quantitative stage through convenience sampling, where all nurses working in their units were invited to participate. They then indicated their interest in proceeding to the second stage, involving semi-structured interviews. 

In this regard, the qualitative stage occurred simultaneously: 1) Among respondents from the quantitative stage already listed in the macro project who agreed to participate in the qualitative phase; 2) Participants were also recruited via institutional e-mail, WhatsApp^®^, and by on-site research agents (nursing faculty, undergraduate students, scientific initiation and postgraduate nursing scholarship holders, as well as individuals from institutions affiliated with the macro project, who were trained and qualified to conduct interviews); 3) Upon acceptance, interviewees were asked to provide recommendations of other nurses. 

Participants who agreed signed the Informed Consent Form (ICF) and participated in interviews in two formats: through video calls using the Google Meet^®^ application and/or in a reserved space at their workplace, both conducted individually. 

It is worth mentioning that there was a standardized instrument for approaching participants, which introduced the researchers to the participants, explained the main project, and the duration of the interviews (20 to 50 min), a time frame that was adhered to. 

Online meetings were held and facilitated by the coordinators of the main project to train data collectors and standardize data collection instruments. These were based on the Manual for Qualitative Data Collection in Care Management, Nursing Leadership, and Practice Environment.

Thus, during meetings with the coordination of the main project and the involved researchers from affiliated educational institutions, a date was set to commence interviews. A pilot test was conducted at the HUF affiliated with the main project, with the participation of a nurse. It is important to highlight that this interview was not included in the count. During the meetings held with the coordination and researchers, discussions were held regarding data saturation to define a date for ending the interviews. 

The option of remote interviews was included because, at the time, we were still experiencing a critical scenario of the COVID-19 pandemic.

### Data collection

The interviews were individual and semi-structured, consisting of nine questions about the changes, leadership strategies, and nursing care management during this pandemic. Regarding participant characterization questions, these were answered by participants at the beginning of the interviews, consisting of: initials of the name; gender; age; level of education; years of professional experience; time working with COVID-19 patients. 

Data were collected by the researchers involved in the main project at each location. The Google Meet^®^ application format allowed for recording of the interviews, and when conducted in person, a recorder or mobile phone in voice recorder mode (mp3) was used. Subsequently, the interviews were fully transcribed by the researchers from each location and concluded when the proposed objectives were achieved, after agreement in a virtual meeting (Google Meet^®^) with all the researchers involved, setting the final date for the conclusion of all interviews in all hospitals. 

### Data analysis

For the analysis of the information obtained in the interviews, the content analysis method by Bardin^24^ was used, comprising the following stages: firstly, the pre-analysis phase, involving a “floating reading” and exploration of the content; secondly, the exploration of the material, which includes a more in-depth reading of the findings, followed by the selection of coding units, leading to the construction of categories; and the third phase involves the treatment of the results obtained through analysis and inference^24^. 

After a thorough reading of the interviews, it was decided to create a table using Microsoft Office 365 Word software, highlighting the nurses’ leadership perceptions in COVID-19, and relate the findings according to Kotter’s concepts and his eight-step change model. This analysis was conducted individually for each HUF, resulting in a Word document for each hospital institution. It’s worth noting that a peer review was conducted by the co-authors, contributing to the quality of this research. 

Additionally, we opted to use the webQDA^®^ software, version 3.0, to strengthen the study. This qualitative research application assists researchers in organizing, editing, and visualizing categories and subcategories, allowing for the orderly storage of information^25^. To preserve the anonymity of the participants, they were identified with the initials of each university they belonged to, followed by a numeral in ascending order, for example: *“UFBA01; UFSC 01; UFRJ 01...”.*

### Ethical aspects

All ethical precepts involving studies with human subjects were adhered to, as mandated by Resolution No. 466/2012 of the Brazilian National Health Council^26^. It is worth noting that the study was approved by the Research Ethics Committee (CEP) of the main project, referring to the main project, under Opinion CAAE: 38912820.3.1001.0121, and also approved by the CEP of the institution proposing this study, with CAAE: 38912820.3.2011.0049, both through the *Plataforma Brasil*.

## Results

Regarding the sample of this study, 139 nurses were included, all working in the care of COVID-19 patients. The age of the participants ranged from 23 to 65 years old, with a mean age of 32 years old. There were 118 female participants (87.41%) and 17 male participants (12.59%). Regarding the length of professional experience, the minimum identified was one year of service, and the maximum was 36 years. Finally, in terms of educational attainment, four participants had doctoral degrees, and there were 36 with master’s degrees. 

Regarding the thematic-categorical analysis of the data emerged in the interviews, four categories were identified: Development of essential elements for leading changes: knowledge, teamwork, communication; Relationship of trust and respect; Team motivation, directing towards the goal; and The complexity of leadership and expanded vision. The data comprising each of these categories are presented below. 

### Development of key elements for leading changes: knowledge, teamwork, communication

In the testimonies below, nurses emphasized the importance of knowledge, both regarding their competencies and skills that constitute nursing care, and their abilities to lead teams. According to them, this knowledge allows everyone to adequately prepare to conduct actions towards achieving common goals. Thus, this category is related to some key elements of Kotter’s model, such as the first stage of sense of urgency, in which knowledge of the competencies and skills necessary for nursing care and team leadership is essential to create awareness of the need for change; the second stage of building coalition, being important to gather people with different sets of skills and knowledge to lead change effectively; and the fourth stage of communicating the vision, ensuring that everyone understands the change objectives and is aligned with them.

*Leading is a process where you learn to develop skills* [...] *You need to be humble to learn from people* [...] *and communication is one of the pillars of leadership. A leader who fails to communicate effectively lacks good leadership, as leadership is not a solitary endeavor. If you lead alone, you are not a leader, you are a boss.* (UFBA 01)

[...] *My role as a leader is always to participate jointly with the team, always be together, and it is not just about motivation, not just about education; we have to exchange knowledge because no one knows everything. It is a gain of knowledge, it is an exchange, and many of my colleagues who are technicians are part of my team* [...]. (UFSC 09)

*There are three essential things for leadership to be effective: knowledge, attitude, and how you apply your work with your team. The way you behave with the team is what makes you a leader. So, facing the pandemic, the point that led to good development in care was knowledge. To become a leader, you have to have knowledge.* (UFAM 09)

*The nurse needs to possess knowledge, understanding of their actions, awareness of their competencies, and familiarity with the specific competencies of the nursing team, as delegation is essential. So, if we do not have this knowledge, we cannot delegate important actions for the provision of this assistance. They also need to have knowledge of routines and patient safety protocols because if they do not, they cannot see that in practice* [...] *they need to have attitude, they need those three characteristics: knowledge, skill, and attitude.* [...]. (UFRN 03)

*I do not know any nurse who is not a leader. If you are in clinical practice, you have to manage that team* [...] *It is increasingly important to know your team better and to have scientific knowledge, which is something that some nurses overlook in terms of knowledge* [...]. (UNIFESP 02)

*I have no problem at all in talking to people, in communicating. This is greatly beneficial for the patient because it enhances our ability to communicate* [...]*. In my team, I have observed that a previously existing communication problem has been resolved, and I actively encourage open communication among technicians, they are free to come to me and talk* [...] *this great articulation, I think it is very positive* [...]. (UFRJ 10)

### Relationship of trust and respect

In this second category, the importance of teamwork emerged again, but now based on a relationship of trust and respect. According to the participants, without these elements, it is difficult for a leader to exercise leadership. It is noteworthy to mention the emphasis on empathy in the speeches, particularly given the pandemic context, where respect and collaboration proved to be fundamental. Thus, it is possible to relate the findings to Kotter’s precepts and his model, because the author highlights these elements of mutual trust and respect. For example, in the second stage of building coalition, trust and respect between the leader and his team are necessary to work more collaboratively and effectively to achieve the common objectives of change.

*Regarding the characteristics of a leader: the first thing is to trust the team they have; to have empathy; to stand by the team; to work with the team to help and be on the front line with the other members* [...] *to be the first to step into a combat line and the last to step out* [...] *you have to fight together, side by side, whether in front or covering each other’s back, while also having a well-prepared emotional intelligence* [...] *Because if the leader panics, it is all over- the team, trust, everything. It is important to trust the technician and convey that trust to them as well. You know, you are there to stay with them.* (UFMT 04)

*In terms of personality traits, I think it is important for them to possess empathy. Empathy allows them to understand the perspective of others, to recognize the level of support the team requires, and to identify any difficulties they may be facing so that we can effectively assist them* [...]. (UFRN 03)

*Leaders are the mirror of their team, if you work together with your technicians, with your colleagues, you will transmit to them that confidence. You have to be good at what you do and you have to respect your colleagues, regardless of their rank.* (UFMS 02)

*I perceive leadership as a more horizontal relationship rather than vertical, while still respecting hierarchies. However, for leadership to be truly understood and embraced, it is crucial to listen to the perspectives of those being led. This approach fosters a relationship of trust and respect.* [...]. (UFBA 07)

[...] *I will transmit confidence to my team that they can count on me. We experienced this profoundly during the COVID-19 situations, as many of our colleagues faced precarious circumstances. Whether due to a sick family member or that they could not see their child, because they had to stay away, they could not hug them, nor kiss them* [...]. (UFSC 09)

### Motivating the team, directing towards the objective 

In this third category, the nurses’ perceptions regarding the attributes of motivating, inspiring, and influencing people are presented. According to them, a leader needs to have the courage to lead, delegate wisely, and fulfill this role. Thus, we can relate this to Kotter’s concepts and his model, highlighting the first stage of creating a sense of urgency and the third stage of communicating the vision. Establishing a sense of urgency to motivate people to act towards the change objectives was essential during COVID-19. A motivated and inspiring leader can communicate clearly and convincingly about the change objectives and the purpose behind it.

[...] *to satisfactorily influence people; the leader must have skills, qualities to motivate their own team* [...] *and thus present the objectives, what are the objectives of being there in that sector, in that organization, what we can do better every day* [...] *to really motivate people, the collaborators, that we are a team, we don not work alone, I, as a nurse, cannot work alone, I have nursing technicians, doctors, psychologists, physiotherapists, everyone, it is a team* [...]. (UFMT 01)

[...] *leadership comes from influencing people, inspiring people, delegating wisely; it comes to charting a course towards a goal. I believe a leader is not a leader because of a position. People will see them as a leader not because of a position, but because of their actions, their demeanor, how they carry themselves.* [...]. (UFPA 02)

*I believe courage is an aspect you need to have a lot of. Courage to be a leader, courage to call out those who need to be spoken to, when necessary, sometimes even to confront patients, I believe you need courage.* (UNIFESP 04)

### The complexity of leadership and expanded vision

The testimonies in this final category reveal the complexity and difficulty of leadership, which, in the case of nursing during the COVID-19 pandemic, proved even more challenging because the obstacles were significant and often concentrated in intensive care units, one of the most critical areas in a hospital. Thus, nurses needed an expanded vision, going beyond theory, and experiencing and adapting to practice daily. We can relate that Kotter indicates that a broad vision is essential to understand and deal with the complex challenges faced by leaders during a crisis, but the difficulty lies in changing people’s behavior.

*Leadership is quite challenging, especially concerning acceptance, both of the service and adherence to norms and routines, which often encounters significant resistance. There is resistance to any change. Therefore, we must adapt and find ways to bring everyone closer together, enabling us to progress. It should be like an exchange. I am not a leader alone; we have to exchange experiences and journey together. There is no point in me trying to impose anything.* [...] *Leadership in nursing is an enormous challenge* [...]. (UFPA 07)

*Leading is a very complex role, especially in an intensive care unit, since the nurse’s role is, in my view, the most important within the ICU today because we are a mix of everything that happens. I believe that being a leader transcends the concept we find in literature; it is an experience rooted in a profoundly positive ethical context that only firsthand experience, viewed from a more humanized perspective, can truly provide* [...]. (UFMT 02)

*I believe that leadership goes beyond just managing the department, looking at the technical aspects, logistics, and structure. I think it requires a holistic view from end to end of the department, every corner, every patient, everything. I think the leader has to develop that perspective. Regarding the patient, it is not just about providing care and assistance; it is about looking at their individual needs, their psychological well-being, and considering the family’s perspective as well.* (UFRJ 05)

*I consider it an essential attribute, so to speak, not only for resolving team conflicts but also for understanding in a broader sense how these conflicts impact care. It is about discerning what I or my team can do to enhance care while concurrently managing patient needs and team dynamics, as well as overseeing work processes. So, I believe it is about being able to see comprehensively everything nursing can do to improve this process, both for patients and for the team and the institution as well.* (UFSM 04)

## Discussion

In the context of COVID-19, constant changes occurred in health care practices, and nurse leaders, who were at the forefront of this confrontation, had their actions highlighted. In this sense, the statements allowed us to understand the perceptions of these professionals about the leadership they exerted, discussing them through the concepts of John Kotter’s reference model of eight stages of change.

Initially, the study addresses essential elements of leadership, such as knowledge, teamwork, and communication. A two-way communication emerged, sensitive to others, especially due to the pandemic scenario, which demanded a diligent and attentive response to the sense of urgency of the situation. A leadership based on knowledge and respectful dialogue between the nurse and their team was highlighted, contributing to the teamwork coordination.

In this sense, according to Kotter’s understanding, leaders need to associate certain abilities, such as accumulating relevant knowledge that contributes to an expanded vision. It is also necessary to develop leadership skills related to vision, communication, and motivation for all phases of a change process^17^ ^),(^ ^27^.

In Taiwan, the eight-stage model of Kotter was used in an International Network of Hospitals and Health Services for the certification of Elderly-Friendly Hospitals, reinforcing that the knowledge and attitudes of employees are essential for successful change. Additionally, the model contributed to employee adherence with a focus on success, and it was important, throughout all stages of the process, to adopt clear communication for the understanding of all involved^28^. 

In Brazil, we see similar results, emphasizing the importance of nurses’ knowledge about COVID-19, its aspects, transmission methods, and ways to combat it. It was identified that the main means of learning used by nurses to deal with this disease were focused on seeking information alone, via the Internet or television, as well as participating in training sessions promoted by their respective health institutions through continuing education. However, according to the study, the basis of learning was fundamentally grounded in the experiences of the individuals involved^29^. 

A similar situation is reinforced in Brazil, highlighting that nurses and other health care professionals acting as managers needed to rigorously base their decisions and urgent actions regarding COVID-19 on scientific evidence and recommendations from competent national and international bodies. The leadership of nurses in the adopted practices was highlighted^30^. 

It is noted, therefore, how much the nurse-leader needs to stay updated regarding their knowledge, both within and outside their work context, needing to broaden their vision. A posture of staying updated will allow them to feel prepared to lead their team and, thus, be able to ensure quality in care. 

The study highlighted the trust and respect between the nurse-leader and their team, which also relates to Kotter’s principles. According to the author^16^ ^)-(^ ^17^, during the exercise of change leadership, good teams learn to operate with trust and emotional involvement, while in less inspiring leadership, trust relies only on one person or on no one at all^16^ ^)-(^ ^17^. 

Kotter also considers it extremely difficult to achieve significant transformations, a fact that, according to him, demands a reinforced task force and precisely values the administrative coalition phase (building a strong administrative commission). For him, there must be trust and respect, because otherwise, a single person who manages lack of trust can destroy the work of an entire team^16^ ^)-(^ ^17^ ^),(^ ^27^. 

Thus, encompassing motivation, inspiration, and courage to assume this leadership role, the study^31^ concluded that, in times as uncertain as the COVID-19 pandemic, leaders need to have resilience to deal with adversities and learn from them. Therefore, they must prepare the institution for learning and avoid a leadership crisis, demonstrating credibility, courage, and compassion. It is also necessary to teach their teams indispensable values such as empathy, trust, transparency, and integrity, for them to understand the need for change. Therefore, it is up to the leader to have the courage to speak the truth and to lead amidst uncertainties^31^.

In the United States of America (USA), research revealed a different reality and warned of the seriousness of scenarios where there is no trust in leaders. Reports showed situations of health care professionals who expressed feelings of pain, frustration, and anger and who needed support from their leaders but did not receive it. Additionally, the lack of safe spaces for open dialogue, lack of transparent communication, and feelings of helplessness in patient care, as they were excluded from discussions. The combination of these attributes had a significant impact on the scope of work of these professionals, contributing to the breakdown of a relationship of trust and even their ability to trust themselves^32^. 

Therefore, Kotter’s concept reinforces that if there is no trust within the team, the entire change process will not flow smoothly^16^ ^)-(^ ^18^. Therefore, the nurse leader needs to be attentive and remain faithful to the goal of promoting teamwork harmoniously, benefiting all involved. 

Finally, the testimonies highlighted the difficulty and complexity of leadership, as well as how challenging it is to exercise it, especially in the nursing field and in the context of confronting COVID-19. Being a leader in these situations can become burdensome. 

Leading changes is an important and difficult task for health care leaders, so many individuals, organizations, or communities prefer to remain in their “comfort zones,” posing a problem for change. Another obstacle is getting people to change their attitudes^28^. Kotter emphasizes that the great challenge for the leader lies in changing people’s behavior, requiring constancy in “seeing-feeling-changing”^17^ ^),(^ ^27^ ^).^ However, it is evident that the COVID-19 pandemic scenario has brought to the forefront in nurse leaders a feeling of the need to adapt to these three aforementioned pillars.

In this sense, research shows that this pandemic brought challenges to nurse leadership; however, it aimed to develop and value others, based on trust and resilience within the team^33^. Corroborating the findings, recent research has highlighted the courage, engagement, and professional self-realization of health care workers in tackling COVID-19. However, in the work of nursing professionals, suffering has significantly increased due to a combination of a constantly changing environment and high demands, emphasizing the hardship and increased workload due to the complexity of patient care^34^. 

Therefore, the study contributes to assisting and strengthening the leadership of nurses in environments of constant change and uncertainty, such as health care settings. Through the constructs of John Kotter and his model of change, which is particularly relevant as a leadership tool to support leaders in organizational change processes^16^, it emphasizes the importance of involving people for the process to occur effectively. And, in the face of a crisis or unexpected scenario like this pandemic, nurse leaders are able to reflect and develop a strengthening teamwork, clearly communicating the necessary changes in health care practices and improving patient care.

Regarding the limitations of the study, the data collection period occurred during still complex times in terms of COVID-19 infection and mortality. Although the vaccination strategy had already begun, it was a delicate moment where most professionals were overwhelmed. For this reason, despite the interviews being previously scheduled, postponements and cancellations ended up occurring. On the other hand, this study enables discussions about leadership understandings in nursing and its results contribute to enhancing the professional practice of nurse leaders. 

## Conclusion

In conclusion, the perceptions of Brazilian nurses’ leadership in university hospitals during the COVID-19 pandemic exhibit characteristics consistent with the John Kotter’s principles. This prompts reflections for the practice of nurse leaders to undertake changes, and even for other health care professionals in leadership positions.

Through the understandings of nurses, the presence of essential elements of leadership is highlighted, which, although challenging, are extremely necessary and empowering for the entire process of change to occur with quality and effectiveness. Some of these elements for leading change involve seeking multiple knowledge sources, which involve an exchange of knowledge among nurse leaders, their nursing team, and other health care professionals. Thus, basing leadership on polished communication, facilitating teamwork through a relationship of trust and respect. Nurses recognized the complexity of leadership, acknowledging it as arduous and difficult, especially in the scenario of the COVID-19 pandemic, and further intensified by resistance to change. 

There is a call for the importance of new research addressing the complexity of leadership in nursing, both for its complexity and to update knowledge, making it more understandable for implementation/application in practice. The growing need to develop this competency in environments characterized by changes and significant impacts is considered.
