# Supplementary material for: Nurses’ perceptions of leadership in Brazilian hospitals during COVID-19 through Kotter’s conception
Source: Rev Lat Am Enfermagem. 2024 Aug 19;32:e4281. doi: 10.1590/1518-8345.7151.4281 (PMC11335068; doi:10.1590/1518-8345.7151.4281)
Supplement: Translation

# Translation: Percepções de enfermeiros sobre liderança em hospitais brasileiros na COVID-19 pela concepção de Kotter*

**DOI:** 10.1590/1518-8345.7151.4282

**Authors:** Patrícia Alves Galhardo Varanda, Gilberto Tadeu Reis da Silva, Simone Coelho Amestoy, Vânia Marli Schubert Backes, Gabriela Marcellino de Melo Lanzoni, Augusto Ferreira Umpiérrez, Naomy Safira Batista da Silva

**Affiliations:** 1 Universidade Federal da Bahia, Escola de Enfermagem, Salvador, BA, Brasil.; 2 Bolsista da Coordenação de Aperfeiçoamento de Pessoal de Nível Superior (CAPES), Brasil.; 3 Universidade Federal do Vale do São Francisco, Departamento de Enfermagem, Petrolina, PE, Brasil.; 4 Universidade Federal de Santa Catarina, Departamento de Enfermagem, Florianópolis, SC, Brasil.; 5 Universidad Católica del Uruguay, Departamento de Enfermagem, Montevideo, Montevideo, Uruguai.

**Keywords:** Enfermagem, Liderança, Enfermeiras e enfermeiros, COVID-19, Gestão de mudança, Pandemias

## Abstract

**Objetivo:**

conhecer as percepções de liderança de enfermeiros durante a pandemia da COVID-19 em hospitais universitários brasileiros, pela concepção dos conceitos do referencial de John Kotter e seu modelo de mudança de oito etapas.

**Método:**

pesquisa multicêntrica de natureza qualitativa e delineamento analítico. A amostra foi constituída por 139 enfermeiros atuantes na COVID-19 entrevistados mediante um roteiro de entrevistas semiestruturadas, categorizadas por meio de análise de conteúdo de Bardin com base nos conceitos de John Kotter e seu modelo de mudança de oito etapas.

**Resultados:**

os achados resultaram em importantes percepções dos enfermeiros acerca da liderança durante o enfrentamento da COVID-19, que apresentaram características condizentes com os preceitos de Kotter. Evidencia-se uma liderança baseada na busca pelo conhecimento, pautada em uma comunicação polida, facilitando o trabalho em equipe por meio de uma relação de confiança e respeito. O reconhecimento da complexidade e dificuldade que é exercer a liderança do enfermeiro, principalmente, em contextos de crise.

**Conclusão:**

as percepções dos enfermeiros reforçam elementos essenciais para a prática da liderança, como a importância pela busca de conhecimentos múltiplos, comunicação polida, relação baseada na confiança e respeito, e o reconhecimento da complexidade em liderar, apresentando, assim, características acerca dos preceitos de Kotter.

## Destaques

**(1)** Evidenciadas percepções de enfermeiros sobre liderança na COVID-19.

**(2)** A COVID-19 intensificou a complexidade no exercício da liderança de enfermeiros.

**(3)** A liderança de enfermeiros foi consolidada no conhecimento, comunicação, confiança.

**(4)** Atuar no enfrentamento da COVID-19 enquanto enfermeiro-líder tornou-se árduo.

**(5)** Preceitos de Kotter auxiliam em reflexões na liderança de enfermeiros.

## Introdução

A liderança é indispensável à prática do enfermeiro, sendo importante que ele busque desenvolvê-la. Embora as definições frente à liderança sejam complexas, reconhecemos uma grande liderança quando a encontramos^1^ ^)-(^ ^2^. Na enfermagem, a liderança pode ser caracterizada como uma competência profissional fundamental no exercício de sua prática clínica e administrativa, na qual o enfermeiro direciona, apoia e orienta sua equipe de saúde, possibilitando alcançar resultados positivos na assistência ao cuidado do paciente^3^ ^)-(^ ^4^.

Os enfermeiros representam o maior grupo de força de trabalho global em saúde, e em algumas situações são os únicos provedores disponíveis^2^. Assim, eles desempenham papéis cruciais na prática, educação e pesquisa em saúde. A Organização Mundial de Saúde (OMS)^5^ ^)-(^ ^6^ enfatiza o fortalecimento da liderança dos enfermeiros para influenciar políticas e decisões de saúde. Destarte, em tempos normais e durante pandemias, os enfermeiros desempenham um papel fundamental na linha de frente do cuidado de saúde e, portanto, contribuindo significativamente para o combate à doença^5^ ^)-(^ ^6^. 

A COVID-19 iniciou em 2019, em Wuhan, na China, conseguindo causar desde um simples resfriado até a síndrome respiratória aguda (*SARS-Cov-2*), conhecida como *Coronavirus Disease 2019* (COVID-19)^7^. Rapidamente, tornou-se uma das piores catástrofes de saúde, impactando os sistemas de saúde globalmente^8^. Em março de 2020, a OMS declarou pandemia de COVID-19. Em 5 de maio de 2023, a OMS anunciou que a COVID-19 não era mais uma emergência de saúde pública, devido à redução nas taxas de internação, admissões em Unidades de Terapia Intensiva (UTIs) e mortalidade mundial^9^ ^)-(^ ^10^.

Contudo, as consequências de uma pandemia são preocupantes e podem ser observadas em diversos setores. Dentre esses, abruptamente, os hospitais foram desafiados a ampliar leitos de terapia intensiva, promover treinamentos de profissionais de saúde para atendimento de pacientes com COVID-19 e adquirir maior quantidade de equipamentos de proteção individual. Lidava-se com um vírus desconhecido, com alto contágio e grande letalidade. Os hospitais universitários federais (HUFs) brasileiros foram elencados como referências de média e alta complexidade do Sistema Único de Saúde (SUS) para assistência de pacientes com COVID-19^9^ ^),(^ ^11^.

No âmbito da gestão, os enfermeiros líderes compuseram comissões com fins de planejamento e rápida organização de fluxos assistenciais, ajustes na infraestrutura das UTIs e elaboração de protocolos relacionados à assistência aos pacientes internados por consequência da COVID-19. Outro elemento de destaque na atuação destes profissionais durante a pandemia foi a articulação entre a equipe multiprofissional, para promover ajustes necessários à promoção de um cuidado holístico, com procedimentos operacionais padronizados, *checklists* de segurança e atualização das orientações de prática clínica, sempre que disponíveis^12^.

Essa pandemia, portanto, ocasionou uma crise mundial e, no contexto da enfermagem, oportunizou destacar a liderança do enfermeiro para seu enfrentamento^13^. Estudo^14^ ^)^ assinalou que os enfermeiros demonstraram forte sentimento de responsabilidade e brio por prestarem os cuidados de enfermagem aos pacientes com COVID-19. Enfermeiros gestores e especialistas desempenharam papéis fundamentais na coordenação durante a pandemia, e a liderança de enfermagem se destacou como elemento crucial nessa manutenção, inclusive, no sentido de manter uma boa relação entre enfermagem e paciente^14^.

Neste contexto de extremas incertezas, a dedicação da enfermagem assumiu um valor imensurável. As habilidades de liderança dos enfermeiros, globalmente, foram colocadas à prova, suas ações mostraram-se essenciais para futuras gerações^15^ ^)^ e ficou notória a necessidade de preparo destes profissionais para lidar com essas situações, que podem, inclusive, acontecer novamente, ainda que por diferentes causas^15^. Dessa forma, estudos que evidenciem elementos capazes de contribuir com o desenvolvimento da liderança por enfermeiros são importantes e prementes, principalmente em cenários de crise, em que esta competência se revela fundamental.

Nesse sentido, foi proposto o presente estudo, que se mostra capaz de fortalecer ainda mais as contribuições deste tipo de pesquisa, por trazer um olhar voltado para os conceitos do referencial teórico de John Kotter e seu modelo de mudança de oito etapas. O autor, reconhecido internacionalmente pelo estudo de liderança e mudanças em organizações, afirma que a liderança é um conjunto de processos intrínsecos das organizações adaptáveis para modificar consideravelmente as circunstâncias, visando ao futuro, alinhando e motivando pessoas^16^. 

Kotter é criador do modelo de “processo de mudança de oito etapas”, conceituado em sua obra *“Leading Change”* (Liderando Mudanças). A adoção de tal modelo permite que o líder não cometa os oito erros mais comuns em um processo de mudança e consiga implementá-la eficazmente. O modelo consiste na oposição a esses oito erros identificados durante as seguintes etapas: Senso de Urgência (razões da mudança); Coalizão Administrativa (agentes da mudança); Visão e estratégia (visão de futuro clara e objetiva); Comunicação (devidamente comunicada); *Empowerment* (empoderamento das pessoas); Conquistas a curto prazo (motivar o progresso); Consolidação dos ganhos (manter o foco); e Novos métodos na cultura (incorporação da mudança)^16^ ^)-(^ ^18^.

O modelo de oito etapas de Kotter é uma ferramenta fortalecedora para os líderes efetuarem mudanças de forma eficaz, que pode ser aplicada ao contexto de saúde. Para tanto, é importante que líderes de enfermagem busquem compreender e aplicar o modelo acerca dos processos de mudança e contribuam com melhorias na qualidade da assistência ao cuidado. Trata-se de etapas interligadas e que se complementam para assegurar uma mudança efetiva, assim, dependendo do cenário, algumas etapas podem acontecer ao mesmo tempo. Desse modo, o autor reforça a importância de não se pular nenhuma etapa do processo de mudança e, quando todos os estágios são bem conduzidos, obtém-se um resultado de sucesso. Porém, em um mundo complexo em que vivemos, alguns casos não seguem rigorosamente o fluxo de oito passos, mas o padrão das oito etapas é o paradigma básico que se observa em processos de mudança bem-sucedidos^16^ ^)-(^ ^18^.

Nesse ínterim, foram encontrados estudos internacionais^19^ ^)-(^ ^21^, na Região Metropolitana da Filadélfia, na África e Reino Unido, que aplicaram o modelo de John Kotter no contexto de saúde mediante líderes de enfermagem e demais profissionais de saúde, durante a pandemia da COVID-19. Emergiram resultados positivos do modelo de mudança de Kotter e seus conceitos de liderança, embora em alguns casos os profissionais tenham necessitado mudar suas crenças, valores, comportamentos e atitudes inerentes, para, dessa maneira, desenvolverem continuamente novas compreensões, possibilitando, assim, a adoção bem-sucedida da mudança pela maioria dos profissionais^19^ ^)-(^ ^21^. Contudo, não foram encontrados estudos no contexto da enfermagem na América Latina. 

Diante do exposto, surgiu o seguinte questionamento: quais as percepções de liderança de enfermeiros durante a pandemia da COVID-19, em hospitais universitários brasileiros, sob os conceitos de John Kotter? Deste modo, para respondê-la, apresentou-se o seguinte objetivo: conhecer as percepções de liderança de enfermeiros durante a pandemia da COVID-19, em hospitais universitários brasileiros, pela concepção dos conceitos do referencial de John Kotter e seu modelo de mudança de oito etapas.

## Método

### Delineamento do estudo

Trata-se de um estudo multicêntrico de natureza qualitativa e delineamento analítico respaldado nos construtos teóricos de John Paul Kotter e seu modelo de oito etapas de mudança. O desenho qualitativo foi considerado o mais adequado para se compreender e identificar as percepções de liderança de enfermeiros no contexto da pandemia. Para assegurar o rigor metodológico do estudo, foram utilizados os itens do *checklist* para pesquisas qualitativas conforme as diretrizes do *Consolidated Criteria For Reporting Qualitative Research* (COREQ)^22^.

### Local do estudo

O estudo foi desenvolvido em dez hospitais universitários brasileiros, os quais são campos práticos para diferentes cursos no contexto de saúde, incluindo a enfermagem. Estes atendem casos de média e alta complexidade para o Sistema Único de Saúde (SUS)^23^, e oito deles são ligados à Empresa Brasileira de Serviços Hospitalares (EBSERH). Tais hospitais estão localizados nas Regiões Sul (dois hospitais, incluindo a instituição matriz); Sudeste (dois); Nordeste (dois hospitais, um dos quais a instituição proponente do pesquisador); Norte (dois); e Centro-Oeste (dois). A escolha por esses dez hospitais universitários se deu, primeiramente, de forma intencional, mediante a interação entre os grupos de pesquisas, docentes e pesquisadores vinculados às instituições de ensino integradas a estes hospitais universitários, assim como por estes representarem as cinco Regiões do Brasil e por terem sido referências no atendimento ao paciente com COVID-19 pelo SUS, durante a pandemia. Deste modo, o primeiro contato com os hospitais universitários se deu pelos docentes que eram referência na coordenação do projeto multicêntrico de cada uma das instituições de ensino.

O estudo foi desenvolvido em dez hospitais universitários brasileiros, os quais são campos práticos para diferentes cursos no contexto de saúde, incluindo a enfermagem. Estes atendem a casos de média e alta complexidade para o Sistema Único de Saúde (SUS)^23^, e oito deles são ligados à Empresa Brasileira de Serviços Hospitalares (EBSERH). 

Tais hospitais estão localizados nas Regiões: Sul (dois hospitais, incluindo a instituição matriz); sudeste (dois); Nordeste (dois hospitais, um dos quais a instituição proponente do pesquisador); norte (dois); e Centro-Oeste (dois). 

A escolha por esses dez hospitais universitários se deu, primeiramente, de forma intencional mediante a interação entre os grupos de pesquisas, docentes e pesquisadores vinculados às instituições de ensino integradas a estes hospitais universitários, assim como por estes representarem as cinco Regiões do Brasil. Além disso, essas são referências no atendimento do paciente com COVID-19 pelo SUS, durante a pandemia. Deste modo, o primeiro contato com os hospitais universitários se deu pelos docentes que eram referências na coordenação do projeto multicêntrico de cada uma das instituições de ensino.

### Período

A coleta de dados ocorreu entre os meses de março de 2021 a abril de 2022.

### População, critérios de seleção, definição da amostra

A amostra se compôs de um total de 139 entrevistados, e os participantes da pesquisa foram enfermeiros atuantes na assistência ao paciente com COVID-19, sem qualquer vínculo empregatício. Constituíram-se critérios de elegibilidade: ser enfermeiro atuante com pacientes com COVID-19, por no mínimo três meses, tempo determinado por se acreditar na possibilidade de se criar uma experiência acerca do fenômeno abordado. 

Cabe salientar que devido a este estudo estar vinculado a um projeto multicêntrico que utilizou métodos mistos, primeiramente, o projeto maior realizou a etapa quantitativa, por meio de uma amostragem por conveniência, em que todos os enfermeiros atuantes em suas unidades foram convidados a participar. E estes respondiam se tinham interesse em seguir para a segunda etapa, mediante entrevistas semiestruturada. 

Nesse sentido, a etapa qualitativa ocorreu concomitantemente: 1) por meio dos respondentes da etapa quantitativa já listados no macroprojeto e que aceitaram participar da fase qualitativa; 2) os participantes também foram recrutados por *e-mail*, *WhatsApp* ^*®*^ institucional, e por agentes da pesquisa *in loco* (docentes de enfermagem, estudantes da graduação, bolsistas de iniciação científica e pós-graduação em Enfermagem e das instituições vinculadas ao macroprojeto, que foram treinados e capacitados para realizarem as entrevistas); 3) mediante o aceite, foram solicitadas aos entrevistados indicações de outros enfermeiros. 

Os participantes que aceitaram assinaram o Termo de Consentimento Livre e Esclarecido (TCLE) e participaram das entrevistas sob dois formatos: por meio de videochamadas realizadas pelo aplicativo *Google Meet* ^*®*^ e/ou em um espaço reservado do local de trabalho dos mesmos, ambos realizados individualmente. 

É válido mencionar que houve um instrumento padronizado para a abordagem aos participantes, o qual apresentava os pesquisadores aos participantes, explicitava sobre o projeto matriz, e duração das entrevistas (20 a 50 min), tempo esse seguido. 

Foram realizadas reuniões *online* e mediadas pelos coordenadores do projeto matriz, para a capacitação dos coletadores e padronização dos instrumentos de coleta de dados, em que foram baseadas no Manual de Coleta Qualitativa frente à Gestão do Cuidado, Liderança de Enfermagem e Ambiente de Prática.

Desde modo, nas reuniões realizadas com a coordenação do projeto matriz e seus pesquisadores envolvidos das instituições de ensino vinculadas, determinou-se uma data para iniciar as entrevistas, sendo realizado um teste-piloto no HUF vinculado ao projeto matriz, em que contou com a participação de um enfermeiro. Destaca-se que essa entrevista não foi contabilizada. Durante as reuniões realizadas com a coordenação e seus pesquisadores foi discutido em conjunto acerca da saturação dos dados para se definir uma data de finalização das entrevistas. 

Incluiu-se a opção da entrevista remota porque, na época, ainda vivenciávamos um cenário crítico da pandemia de COVID-19. 

### Coleta de dados

As entrevistas foram individuais e semiestruturadas, compostas por nove questões acerca das mudanças, estratégias de liderança e gestão do cuidado de enfermagem durante o enfrentamento desta pandemia. Em face das questões de caracterização dos participantes, essas foram respondidas pelos participantes no início das entrevistas, sendo compostas pelas questões: iniciais do nome; sexo; idade; nível de formação; tempo de atuação profissional; tempo de atuação ao paciente com COVID-19. 

Os dados foram coletados pelos pesquisadores envolvidos no projeto matriz de cada localidade. O formato do aplicativo *Google Meet* ^*®*^ permitiu realizar a gravação das entrevistas e, quando presenciais, utilizou-se um gravador ou celular na função de gravador de voz (mp3). Em seguida, as entrevistas foram transcritas integralmente pelos pesquisadores de cada localidade e finalizadas quando alcançados os objetivos propostos, após acordo em reunião virtual (*Google Meet* ^*®*^ ) com todos os pesquisadores envolvidos, delimitando a data final para o encerramento de todas as entrevistas em todos os hospitais. 

### Análise dos dados

Para a análise das informações obtidas nas entrevistas, utilizou-se o método de análise de conteúdo de Bardin^24^, com as seguintes fases: primeiramente a pré-análise, em que se realizou uma “leitura flutuante” e a exploração do conteúdo; em segundo, a exploração do material, em que há leitura mais intensa dos achados, seguida da escolha das unidades de codificação, ocorrendo a construção das categorias; e a terceira fase é o tratamento dos resultados obtidos mediante análise e inferência^24^. 

Para tanto, após uma leitura minuciosa das entrevistas, optou-se por elaborar uma tabela no programa *Word* da *Microsoft Office 365*, na qual se destacaram as percepções de liderança dos enfermeiros atuantes na COVID-19, e relacionar os achados conforme os conceitos e o modelo de oito etapas de mudança de Kotter. Sendo que essa análise foi realizada individualmente para os HUFs, existindo, portanto, um documento em *Word* para cada uma das instituições hospitalares. É válido sinalizar que foi realizada uma revisão por pares pelos coautores, contribuindo para a qualidade dessa pesquisa.

Optou-se também pelo uso do *software webQDA* ^*®*^ , versão 3.0, na busca de fortalecer o estudo, por ser um aplicativo para pesquisas qualitativas que auxilia o pesquisador nas etapas de organização, edição e visualização das categorias e subcategorias, por permitir o armazenamento ordenado das informações^25^. Para preservar o anonimato dos participantes, estes foram identificados com as iniciais de cada universidade a que pertenciam, seguidas de um numeral em ordem crescente, por exemplo: *“UFBA01; UFSC 01; UFRJ 01...”.*

### Aspectos éticos

Foram atendidos todos os preceitos éticos que envolvem estudos com seres humanos, conforme determina a Resolução n.º 466/2012 do Conselho Nacional de Saúde do Brasil^26^. Salienta-se que o estudo teve a aprovação pelo Comitê de Ética em Pesquisa (CEP) do macroprojeto, referente ao projeto matriz, sob Parecer CAAE: 38912820.3.1001.0121, e também aprovação do CEP da instituição proponente deste estudo, com o CAAE: 38912820.3.2011.0049, ambos por meio da Plataforma Brasil.

## Resultados

Em relação à amostra deste estudo, foram incluídos 139 enfermeiros, todos atuantes no atendimento a pacientes com COVID-19. A idade dos participantes manteve-se na faixa entre 23 anos e 65 anos, com média de 32 anos. Eram do sexo feminino 118 participantes (87,41%) e 17 do masculino (12,59%). Quanto ao tempo de experiência profissional, o mínimo identificado foi de um ano de serviço e o máximo, de 36 anos. Por fim, no que diz respeito ao nível de titulação, quatro participantes possuíam doutorado e havia 36 com mestrado. 

No que se refere à análise temático-categorial dos dados emergidos nas entrevistas, foram identificadas quatro categorias: Desenvolvimento de elementos essenciais para liderar mudanças: conhecimento, trabalho em equipe, comunicação; Relação de confiança e respeito; Motivação da equipe, direcionando para o objetivo; e A complexidade da liderança e visão ampliada. Os dados componentes de cada uma delas são apresentados a seguir. 

### Desenvolvimento de elementos essenciais para liderar mudanças: conhecimento, trabalho em equipe, comunicação

Nos depoimentos abaixo, os enfermeiros destacaram a importância do conhecimento, tanto acerca de suas competências e habilidades que compõem o cuidado de enfermagem, quanto de suas capacidades de liderar as equipes. Segundo eles, este conhecimento permite que todos se preparem para conduzir adequadamente ações em prol do alcance de objetivos em comum. Assim, essa categoria está relacionada a alguns elementos-chave do modelo de Kotter, como a primeira etapa de senso de urgência no qual o conhecimento das competências e habilidades necessárias para o cuidado de enfermagem e liderança de equipes é fundamental para criar uma conscientização sobre a necessidade de mudança; a segunda etapa de coalizão sendo importante reunir pessoas com diferentes conjuntos de habilidades e conhecimentos para liderar a mudança eficazmente; a quarta etapa de comunicar a visão assegurando que todos compreendam os objetivos da mudança e estejam alinhados com eles.

*Liderar é um processo no qual você aprende a desenvolver habilidade* [...] *Você precisa ter humildade para ir aprendendo com as pessoas* [...] *e a comunicação é um dos pilares mesmo da liderança. Um líder que não se comunica não tem uma liderança boa, porque você não faz uma liderança sozinho. Se você faz uma liderança sozinho, você não é líder, você é chefe.* (UFBA 1)

[...] *minha condição como liderança é sempre coparticipar com a equipe, sempre estar junto, e não é só no motivacional, também não apenas no educacional; temos que trocar conhecimento, pois ninguém sabe de tudo. É um ganho de conhecimentos, é uma troca, e muitos colegas meus que são técnicos, fazem parte da minha equipe* [...]*.* (UFSC 09)

*Existem três coisas essenciais para que a liderança seja eficiente: conhecimento, postura e a outra é o que você faz com o seu trabalho com sua equipe. A maneira como você se comporta com a equipe é o que torna você um líder. Então, diante da pandemia, o ponto que levou ao bom desenvolvimento no cuidado foi o conhecimento. Para se tornar um líder, tem que ter conhecimento.* (UFAM 09)

*O enfermeiro precisa ter conhecimento, conhecimento das suas ações, conhecimento das suas competências, da competência específica da equipe de enfermagem, porque a gente precisa saber delegar. Então, se a gente não tem esse conhecimento, não consegue delegar as ações importantes para o exercício dessa assistência. Ele precisa ter conhecimento também das rotinas, dos protocolos de segurança do paciente, porque, se ele não conhece, não consegue enxergar isso na prática* [...] *ele precisa ter atitude, aquelas três características: conhecimento, habilidade e atitude* [...]. (UFRN 03)

*Não conheço nenhum enfermeiro que não seja líder. Eu não conheço, se você está na prática clínica e está ali, você tem que gerenciar aquela equipe* [...] *Cada vez mais conhecer sua equipe, cada vez mais ter conhecimento científico, o qual é algo que alguns enfermeiros esquecem da parte do conhecimento* [...]. (UNIFESP 02)

*Não tenho problema nenhum em conversar com as pessoas, em me comunicar. Isso faz um bem muito grande para o paciente, porque a gente se comunica* [...]*. Na minha equipe, vejo isso, que é um problema que existia e que hoje não existe mais, essa questão de comunicação, e também dou essa liberdade aos técnicos, chegarem para mim e conversar[...]essa grande articulação, acho que está muito boa* [...]. (UFRJ 10)

### Relação de confiança e respeito

Nessa segunda categoria, emergiu novamente a importância do trabalho em equipe, mas agora baseado em uma relação de confiança e respeito. Segundo os participantes, sem esses elementos, dificilmente um líder conseguirá exercer a liderança. Vale destacar, ainda, a menção à empatia nas falas, sobretudo, diante desse cenário de pandemia, no qual o respeito e a colaboração foram fundamentais. Desse modo, é possível relacionar os achados com os preceitos de Kotter e seu modelo, devido ao autor destacar esses elementos de confiança e respeito mútuos. Por exemplo, na segunda etapa de coalizão, são necessários a confiança e o respeito entre o líder e sua equipe para trabalharem de forma mais colaborativa e eficaz para alcançar os objetivos comuns da mudança.

*Em relação às características de um líder: a primeira coisa é confiar na equipe que ele tem; ter empatia; vestir a camisa com a equipe; trabalhar com a equipe para ajudar e estar na linha de frente com os demais* [...] *ser o primeiro a colocar o pé em uma linha de combate e o último a tirar* [...] *tem que estar na batalha junto, de mão dada, ou na frente, ou dando retaguarda atrás, ter uma inteligência emocional bem preparada* [...] *porque, se o líder desesperou, acabou, acabou a equipe, acabou tudo, acabou a confiança; ter a confiança no técnico e passar a confiança para ele também. Você sabe, você está ali para ficar com ele.* (UFMT 04)*.*

*Em relação aos atributos da personalidade, acredito que ele tem que ter empatia, empatia para saber se colocar no lugar do outro, entender até que ponto a equipe está precisando de apoio, se ela está com dificuldade, de que forma a gente vai conseguir auxiliar essa equipe* [...]. (UFRN 03)

*O líder é o espelho da sua equipe, se você trabalha com o seu técnico, com o seu outro colega, você vai transmitir a ele aquela segurança. Você tem que ser bom no que você faz e tem que respeitar os seus colegas, independentemente de qual grau estes tenham.* (UFMS 02)

*Acho que é uma relação mais horizontal que verticalizada, não deixando de respeitar as hierarquias, mas a liderança, para que seja entendida e compreendida, precisa ouvir o lado do liderado, porque aí é que se estabelece uma relação de confiança e respeito.* [...]. (UFBA 07)

[...]*Vou transmitir confiança a minha equipe que pode contar comigo. Então, isso a gente sentiu bastante nessas situações de COVID-19, porque muitas pessoas, muitos colegas, chegavam numa condição muito precária ou por que um familiar ficou doente, ou porque não pode ver o filho, porque tem que ficar afastado, não pode abraçar, não pode beijar* [...]. (UFSC 09)

### Motivação da equipe, direcionando para o objetivo

Nesta terceira categoria, são apresentadas as percepções dos enfermeiros em relação aos atributos de motivar, inspirar e influenciar pessoas. Para eles, um líder precisa ter coragem para liderar, delegar com sabedoria e exercer esse papel. Assim, podemos relacionar com os conceitos de Kotter e seu modelo, destacando a primeira etapa de senso de urgência e a terceira etapa de comunicação da visão. Diante da importância de estabelecer um senso de urgência para motivar as pessoas a agirem em direção aos objetivos da mudança, o que foi essencial durante a COVID-19. Um líder motivado e inspirador consegue se comunicar de forma clara e convincente sobre os objetivos da mudança e o propósito por trás dela.

[...] *influenciar satisfatoriamente as pessoas; o líder tem que ter habilidades, qualidades para motivar a sua equipe mesmo* [...] *e apresentar assim os objetivos, quais os objetivos de estar ali naquele setor, naquela organização, o que a gente pode fazer de melhor a cada dia* [...]*motivar mesmo as pessoas, os colaboradores, que somos uma equipe, não trabalhamos sozinhos, eu, como enfermeira, não tenho como trabalhar sozinha, tenho técnico de enfermagem, tem o médico, tem psicólogo, tem fisioterapeuta, tem todo mundo, é uma equipe.*[...]. (UFMT 01)

[...] *liderança vem de influenciar pessoas, de inspirar pessoas, delegar com sabedoria; ela vem para traçar direção para um objetivo. Acho que o líder não é líder por causa de um cargo, as pessoas vão, digamos assim, as pessoas vão vê-lo como líder não por causa de um cargo, mas por conta das atitudes, da postura de como ele se porta* [...]. (UFPA 02)

*Acredito que coragem, coragem é um aspecto que você precisa ter muito. Coragem para ser líder, coragem para chamar quem precisa ser chamado para conversar, quando precisa, às vezes até para enfrentar pacientes, acredito que você precisa de coragem.* (UNIFESP 04)

### A complexidade da liderança e visão ampliada

Os depoimentos nesta última categoria revelam a complexidade e a dificuldade em liderar, o que, no caso da enfermagem durante a pandemia da COVID-19, revelou-se ainda mais desafiador, pois os obstáculos eram grandes e, muitas vezes, concentrados nas unidades de terapia intensiva, uma das mais críticas em um hospital. Assim, o enfermeiro necessitou de uma visão ampliada, indo além da teoria e vivenciando e adequando a prática, diariamente. Podemos relacionar que Kotter sinaliza que uma visão ampliada é essencial para entender e lidar com os desafios complexos enfrentados pelos líderes diante de uma crise, mas que o árduo está em mudar o comportamento das pessoas.

*Liderança é muito difícil, a questão da aceitação, tanto do serviço como de seguir normas e rotinas, apresenta muita resistência. São resistências a qualquer mudança. Então, a gente tem que se adaptar, encontrar maneiras de trazê-los para perto, para podermos prosseguir. Tem que ser como uma troca. Eu não sou líder sozinha, a gente tem que trocar experiências e andar juntos, não adianta eu querer impor nada.* [...] *Liderança na Enfermagem é um desafio enorme* [...]. (UFPA 07)

*Liderar é uma função bem complexa, acho que principalmente numa unidade de terapia intensiva, uma vez que a figura do enfermeiro é, a meu ver, o papel mais importante dentro da UTI hoje, porque a gente é um misto de tudo o que acontece. Acho que ser líder vai muito além do conceito que a gente pega nas literaturas, acho que é uma vivência que tem que vir de um contexto ético bem positivo que somente a vivência mesmo, em uma perspectiva de um olhar mais humanizado* [...]. (UFMT 02)

*Acredito que a liderança vai além de liderar o setor, de ver a questão técnica, a logística, a estrutura. Acho que tem que ter um olhar holístico de ponta a ponta do setor, todo cantinho, cada paciente, cada coisa, acho que o líder tem que desenvolver esse olhar. Em relação ao paciente, não só em relação ao cuidado, prestar assistência e ter um olhar na particularidade dele, na questão psicológica, na questão de pensar no familiar* [...]. (UFRJ 05)

*Acredito que é um atributo, digamos assim, que precisamos ter, no sentido não só de resolução de conflitos da equipe, mas de enxergar também de forma ampliada como que isso implica o cuidado e o que eu posso fazer ou o que a minha equipe pode fazer, para melhorar o cuidado, ao mesmo tempo em que cuido dos pacientes e da equipe e cuidar dos processos de trabalho. Então, acredito que é nesse sentido de conseguir enxergar de forma ampla tudo o que a enfermagem pode fazer para melhorar esse processo, tanto para os pacientes quanto para a própria equipe e para a instituição.* (UFSM 04)

## Discussão

No contexto da COVID-19, constantes mudanças ocorreram nas práticas assistenciais em saúde, e os enfermeiros-líderes, que estiveram na linha de frente desse enfrentamento, tiveram sua atuação evidenciada. Nesse sentido, as falas permitiram conhecer as percepções desses profissionais acerca da liderança por eles exercida, discutindo-se mediante as concepções dos conceitos do referencial de John Kotter e seu modelo de mudança de oito etapas.

Primeiramente, o estudo aborda elementos essenciais de liderança, como conhecimento, trabalho em equipe e comunicação. Emergiu uma comunicação por via de mão dupla e sensível ao outro, especialmente, em virtude do cenário da pandemia, que demandava uma resposta diligente e atenta ao senso de urgência da situação. Destacou-se uma liderança baseada em conhecimento e no diálogo respeitoso entre o enfermeiro e sua equipe, contribuindo com a articulação do trabalho em equipe.

Nesse sentido, mediante os entendimentos de Kotter, os líderes necessitam associar certas capacidades, tais como a de acumular conhecimentos relevantes que contribuam para uma visão ampliada. Necessário também desenvolver habilidades de liderança relacionadas à visão, à comunicação e à motivação para todas as fases de um processo de mudança^17^ ^),(^ ^27^.

Em Taiwan, utilizou-se o modelo de oito etapas de Kotter em uma Rede Internacional de Hospitais e Serviços de Saúde para a certificação de Hospital Amigo do Idoso, e reforçou que o conhecimento e atitudes dos funcionários são essenciais para uma mudança bem-sucedida. Além disso, o modelo contribuiu para a adesão dos funcionários com foco no sucesso, tendo sido importante, durante todas as etapas do processo, adotar uma comunicação clara para o entendimento de todos os envolvidos^28^. 

No Brasil, vemos resultados semelhantes e ressalta-se a importância do conhecimento dos enfermeiros acerca da COVID-19, seus aspectos, forma de transmissão e maneiras de combatê-la. Identificou-se que os principais meios de aprendizagem utilizados pelos enfermeiros para lidar com esta doença concentraram-se na busca de informações sozinhos, via Internet ou televisão, bem como participar de capacitações promovidas pelas respectivas instituições de saúde por meio da educação permanente. Contudo, ainda segundo o estudo, a base do aprendizado esteve fundamentada nas vivências dos sujeitos envolvidos^29^. 

Reforça-se situação semelhante, no Brasil, destacando que os enfermeiros e demais profissionais da saúde, atuantes como gestores, necessitaram se embasar rigorosamente nas evidências científicas e recomendações dos órgãos competentes mundiais e nacionais para a tomada de decisões e ações urgentes acerca da COVID-19. Evidenciou-se o protagonismo dos enfermeiros frente às condutas adotadas^30^. 

Nota-se, portanto, o quanto o enfermeiro-líder precisa estar atualizado em relação a seus conhecimentos, tanto dentro como fora do seu contexto de trabalho, necessitando ampliar sua visão. Uma postura de manter-se atualizado permitirá, inclusive, que ele se sinta preparado para conduzir sua equipe e, assim, apto a assegurar a qualidade na assistência. 

O estudo destacou a confiança e o respeito entre o enfermeiro-líder e sua equipe, o que também se relaciona com os preceitos de Kotter. Segundo o autor^16^ ^)-(^ ^17^, durante o exercício da liderança da mudança, boas equipes aprendem a operar com confiança e envolvimento emocional, enquanto em uma liderança menos inspiradora, confia apenas em uma única pessoa ou em ninguém^16^ ^)-(^ ^17^. 

Kotter ainda considera extremamente árduo realizar grandes transformações, fato que, segundo ele, demanda uma força tarefa reforçada e valoriza justamente a fase da coalizão administrativa (criação de uma forte comissão administrativa). Para ele, deve haver confiança e respeito, pois, do contrário, uma única pessoa que gere falta de confiança consegue destruir o trabalho de uma equipe inteira^16^ ^)-(^ ^17^ ^),(^ ^27^. 

Desse modo, englobando a motivação, a inspiração e a coragem para assumir esse papel de líder, estudo^31^ concluiu que, em tempos tão incertos como o da pandemia pela COVID-19, os líderes necessitam ter resiliência para lidar com as adversidades e aprender com elas. Para tanto, devem preparar a instituição para o aprendizado e evitar uma crise da liderança, demonstrando credibilidade, coragem e compaixão. Necessário ainda que ensinem às suas equipes valores imprescindíveis, como empatia, confiança, transparência e integridade, para entenderem a necessidade das mudanças. Para tanto, cabe ao líder coragem para falar a verdade e liderar em meio às incertezas^31^.

Nos Estados Unidos da América (EUA), pesquisa evidenciou uma realidade distinta e alertou para a gravidade de cenários em que não há confiança nos líderes. Os relatos evidenciaram situações de profissionais de saúde que demonstraram sentimentos de dor, frustrações e raiva e que precisavam do apoio de seus líderes e não o tiveram. Além disso, ausência de espaços seguros para um diálogo aberto, falta de uma comunicação transparente e sentimento de impotência no cuidado do paciente, por ficarem de fora de discussões. A soma desses atributos exerceu significativo impacto no escopo de trabalho desses profissionais, contribuindo para o rompimento de uma relação de confiança e até mesmo da capacidade de confiarem em si^32^. 

Destarte, o conceito de Kotter reforça que, se não houver confiança entre a equipe, todo o processo de mudança não fluirá^16^ ^)-(^ ^18^. Por isso, o enfermeiro-líder necessita estar atento e manter-se fiel ao objetivo de promover o trabalho em equipe de forma harmônica, no sentido de beneficiar todos os envolvidos. 

Por fim, os depoimentos destacaram a dificuldade e a complexidade da liderança, bem como o quanto é árduo exercê-la, sobretudo, no âmbito da enfermagem e no contexto de enfrentamento da COVID-19. Ser líder, nessas situações, pode se tornar penoso. 

Liderar mudanças é uma tarefa importante e difícil para os líderes de saúde, de modo que muitos indivíduos, organizações ou comunidades preferem permanecer em suas “zonas de conforto”, constituindo um problema para a mudança. Outro obstáculo é conseguir que as pessoas mudem suas atitudes^28^. Kotter salienta que o grande desafio para o líder está em mudar o comportamento das pessoas, sendo necessária uma constância em “ver-sentir-mudar”^17^ ^),(^ ^27^. Contudo, percebe-se que o cenário da pandemia da COVID-19 aflorou nos enfermeiros-líderes um sentimento de ser necessário se adequar a esses três pilares supramencionados.

Nesse sentido, uma pesquisa mostra que essa pandemia trouxe desafios para a liderança do enfermeiro, porém, este teve em vista desenvolver e valorizar o outro, baseado na confiança e resiliência entre a equipe^33^. Corroborando os achados, uma pesquisa recente evidenciou a coragem, o envolvimento e a autorrealização profissional dos trabalhadores de saúde para o enfrentamento da COVID-19. Porém, no trabalho dos profissionais de enfermagem, aumentou significativamente o sofrimento devido à soma de um cenário em constantes mudanças e altas exigências, assim como destacando a penosidade e o aumento de trabalho pela complexidade nos cuidados dos pacientes^34^. 

Desse modo, o estudo contribui para auxiliar e fortalecer a liderança dos enfermeiros em ambientes de constantes mudanças e incertezas, como o da saúde. Por meio dos constructos de John Kotter e seu modelo de mudanças, por ser particularmente uma ferramenta de liderança relevante para apoiar os líderes nos processos de mudanças das organizações^16^, enfatizando a importância de envolver as pessoas para o processo ocorrer de maneira eficaz. E, diante de um cenário de crise ou inesperado como o desta pandemia, os enfermeiros-lideres consigam refletir e desenvolver um fortalecedor trabalho em equipe, comunicando com clareza as mudanças necessárias nas práticas do cuidado em saúde e melhorando a assistência ao cuidado.

Quanto às limitações do estudo, o período da coleta de dados ocorreu em momentos ainda complexos em termos de infecção e mortalidade por COVID-19. Embora a estratégia de vacinação já tivesse sido iniciada, tratava-se de um momento delicado, em que, na maioria, os profissionais estavam sobrecarregados. Por esse motivo, ainda que as entrevistas tenham sido previamente agendadas, adiamentos e desistências acabaram ocorrendo. Em contrapartida, este estudo possibilita discussões acerca dos entendimentos de liderança no âmbito da enfermagem, e seus resultados contribuem para qualificar o exercício profissional do enfermeiro-líder. 

## Conclusão

Conclui-se que as percepções de liderança de enfermeiros brasileiros atuantes em hospitais universitários, durante a pandemia da COVID-19, apresentam características condizentes com os preceitos de John Kotter. Isso suscita reflexões para a prática de enfermeiros-líderes para empreenderem mudanças, e até mesmo para outros profissionais da saúde que ocupem cargos de liderança.

Destaca-se, por meio dos entendimentos dos enfermeiros, a presença de elementos essenciais à liderança que, embora desafiadores, são extremamente necessários e fortalecedores para que todo o processo de mudança ocorra com qualidade e eficácia. Alguns desses elementos para liderar as mudanças referem-se a uma busca de conhecimentos múltiplos, os quais envolvem uma troca de saberes entre o enfermeiro-líder, sua equipe de enfermagem e demais profissionais da saúde. Assim, pautando-se em uma liderança baseada na comunicação polida, facilitando o trabalho em equipe por meio de uma relação de confiança e respeito. Os enfermeiros reconheceram a complexidade de liderar, sendo isso árduo e difícil, principalmente no cenário da pandemia de COVID-19, e intensificado pela resistência às mudanças. 

Alerta-se para a importância de novas pesquisas que abordem a temática da liderança na enfermagem, tanto pela complexidade da mesma, como para atualizar o conhecimento e torná-lo mais compreensível para implementar/aplicar à prática. Considera-se a crescente necessidade de desenvolvimento desta competência em cenários de mudanças e de grandes impactos.

# Translation: Percepciones de los enfermeros sobre el liderazgo en hospitales brasileños durante la pandemia de COVID-19 según la concepción de Kotter*

**DOI:** 10.1590/1518-8345.7151.4280

**Authors:** Patrícia Alves Galhardo Varanda, Gilberto Tadeu Reis da Silva, Simone Coelho Amestoy, Vânia Marli Schubert Backes, Gabriela Marcellino de Melo Lanzoni, Augusto Ferreira Umpiérrez, Naomy Safira Batista da Silva

**Affiliations:** 1 Universidade Federal da Bahia, Escola de Enfermagem, Salvador, BA, Brasil.; 2 Becaria de la Coordenação de Aperfeiçoamento de Pessoal de Nível Superior (CAPES), Brasil.; 3 Universidade Federal do Vale do São Francisco, Departamento de Enfermagem, Petrolina, PE, Brasil.; 4 Universidade Federal de Santa Catarina, Departamento de Enfermagem, Florianópolis, SC, Brasil.; 5 Universidad Católica del Uruguay, Departamento de Enfermagem, Montevideo, Montevideo, Uruguai.

**Keywords:** Enfermería, Liderazgo, Enfermeras y enfermeros, COVID-19, Gestión del Cambio, Pandemias

## Abstract

**Objetivo:**

comprender las percepciones sobre liderazgo de los enfermeros durante la pandemia de COVID-19 en hospitales universitarios brasileños, a través de la concepción de los conceptos del marco de John Kotter y su modelo de cambio de ocho pasos.

**Método:**

investigación multicéntrica de carácter cualitativo y diseño analítico. La muestra estuvo compuesta por 139 enfermeros que trabajaban durante la pandemia de COVID-19 entrevistados mediante una guía de entrevistas semiestructuradas; categorizadas a través del análisis de contenido de Bardin basándose en los conceptos de John Kotter y su modelo de cambio de ocho pasos.

**Resultados:**

los hallazgos revelaron importantes percepciones de los enfermeros sobre el liderazgo en la lucha contra el COVID-19, que presentó características consistentes con los preceptos de Kotter. Se observa un liderazgo basado en la búsqueda del conocimiento y una comunicación respetuosa, que facilita el trabajo en equipo por medio de una relación de confianza y respeto. Se reconoce lo complejo y difícil que es ejercer el liderazgo como enfermero, especialmente en contextos de crisis.

**Conclusión:**

las percepciones de los enfermeros destacan elementos esenciales para la práctica del liderazgo, como la importancia de adquirir múltiples conocimientos, tener una comunicación respetuosa, relaciones basadas en la confianza y el respeto, y reconocer la complejidad del liderazgo, que representa las características de los preceptos de Kotter.

## Destacados

**(1)** Se señalan las percepciones de los enfermeros sobre el liderazgo durante la pandemia de COVID-19.

**(2)** El COVID-19 aumentó la complejidad del ejercicio del liderazgo de los enfermeros.

**(3)** Se consolidó el liderazgo de los enfermeros en conocimiento, comunicación, confianza.

**(4)** Trabajar para combatir el COVID-19 como enfermero líder fue arduo.

**(5)** Los preceptos de Kotter ayudan a reflexionar sobre el liderazgo de los enfermeros.

## Introducción

El liderazgo es esencial para la práctica de los enfermeros y es importante que traten de desarrollarla. Aunque las definiciones de liderazgo son complejas, reconocemos un gran liderazgo cuando lo vemos^1^ ^)-(^ ^2^. En enfermería, el liderazgo puede caracterizarse como una competencia profesional fundamental para el ejercicio de la práctica clínica y administrativa, en la que el enfermero dirige, apoya y orienta a su equipo de salud, lo que posibilita lograr resultados positivos en la atención al paciente^3^ ^)-(^ ^4^.

Los enfermeros representan la mayor fuerza de trabajo mundial en salud y, en ciertas situaciones, son los únicos trabajadores disponibles^2^. Por ello, desempeñan papeles cruciales en la práctica, la educación y la investigación en salud. La Organización Mundial de la Salud (OMS)^5^ ^)-(^ ^6^ destaca que hay que fortalecer el liderazgo de los enfermeros para que influyan en las políticas y decisiones de salud. Por lo que, en tiempos normales y durante las pandemias, el enfermero juega un papel fundamental en la primera línea de la atención de salud y, por lo tanto, contribuye significativamente en la lucha contra la enfermedad^5^ ^)-(^ ^6^.

El COVID-19 comenzó en 2019, en Wuhan, China, causando desde un simple resfriado hasta el síndrome respiratorio agudo (SARS-Cov-2), conocido como *Coronavirus Disease 2019* (COVID-19)^7^. Rápidamente se convirtió en una de las peores catástrofes sanitarias y afectó los sistemas de salud a nivel mundial^8^. En marzo de 2020, la OMS declaró al COVID-19 pandemia. El 5 de mayo de 2023, la OMS anunció que el COVID-19 ya no era una emergencia de salud pública, debido a que disminuyeron las tasas de hospitalización, los ingresos a las Unidades de Cuidados Intensivos (UCI) y la mortalidad a nivel mundial^9^ ^)-(^ ^10^.

Sin embargo, las consecuencias de una pandemia son preocupantes y se pueden observar en diferentes sectores. Entre los que se encuentran los hospitales, que se vieron abruptamente desafiados a ampliar las camas de cuidados intensivos, promover la capacitación de los profesionales de la salud para atender a pacientes con COVID-19 y adquirir una mayor cantidad de equipos de protección personal. Lidiábamos con un virus desconocido, de alto contagio y gran letalidad. Los hospitales universitarios federales (HUF) brasileños fueron catalogados como hospitales de referencia de mediana y alta complejidad del Sistema Único de Salud (SUS) para la atención de pacientes con COVID-19^9^ ^),(^ ^11^.

En el ámbito de la gestión, los enfermeros líderes formaron comités con el objetivo de planificar y organizar rápidamente los flujos de atención, realizar adecuaciones a la infraestructura de la UCI y elaborar protocolos relacionados con la atención de los pacientes hospitalizados como consecuencia del COVID-19. Otro elemento destacable en la actuación de estos profesionales durante la pandemia fue la coordinación del equipo multidisciplinario, para promover los ajustes necesarios para brindar una atención integral, con procedimientos operativos estandarizados, *checklists* de seguridad y actualización de guías de práctica clínica, cuando estaban disponibles^12^.

Por lo tanto, esa pandemia provocó una crisis mundial y, en el contexto de la enfermería, permitió destacar el liderazgo de los enfermeros para enfrentarla^13^. Un estudio^14^ indicó que los enfermeros demostraron un fuerte sentimiento de responsabilidad y orgullo por brindar cuidados de enfermería a los pacientes con COVID-19. Los enfermeros gestores y especialistas desempeñaron papeles fundamentales en la coordinación durante la pandemia, y el liderazgo de enfermería se destacó como elemento crucial para mantenerla, incluso para mantener una buena relación enfermero-paciente^14^.

En este contexto de extrema incertidumbre, la dedicación de los enfermeros adquirió un valor inconmensurable. Las habilidades de liderazgo de los enfermeros, a nivel mundial, fueron puestas a prueba, sus acciones resultaron esenciales para las generaciones futuras^15^ y quedó claro que es necesario preparar a esos profesionales para afrontar ese tipo de situaciones, que incluso podrían volver a ocurrir por diferentes causas^15^. Por ende, es importante y urgente que se realicen estudios que destaquen los elementos que pueden contribuir al desarrollo del liderazgo del enfermero, especialmente en escenarios de crisis, en los que esta competencia es fundamental.

Por lo tanto, se propuso el presente estudio, que es capaz de fortalecer aún más las contribuciones a este tipo de investigaciones, al adoptar una perspectiva basada en los conceptos del marco teórico de John Kotter y su modelo de cambio de ocho pasos. El autor, reconocido internacionalmente por su estudio sobre el liderazgo y los cambios en las organizaciones, afirma que el liderazgo es un conjunto de procesos intrínsecos en las organizaciones que pueden adaptarse para modificar considerablemente las circunstancias, apuntando al futuro, convocando y motivando a las personas^16^. 

Kotter es el creador del modelo del “proceso de cambio de ocho pasos”, conceptualizado en su obra “*Leading Change*” (Liderar el Cambio). Adoptar un modelo de este tipo le permite al líder evitar cometer los ocho errores más comunes en un proceso de cambio y poder implementarlo de manera efectiva. El modelo consiste en contrarrestar estos ocho errores identificados durante los siguientes pasos: Sentido de Urgencia (razones del cambio); Coalición Administrativa (agentes del cambio); Visión y estrategia (visión clara y objetiva del futuro); Comunicación (debidamente comunicada); Empoderamiento (empoderar a las personas); Logros a corto plazo (motivar el progreso); Consolidación de ganancias (mantener el enfoque); y Nuevos métodos en la cultura (incorporar el cambio)^16^ ^)-(^ ^18^.

El modelo de ocho pasos de Kotter es una herramienta que fortalece a los líderes para que efectúen cambios de manera efectiva y se puede aplicar al contexto de la salud. Para ello, es importante que los líderes de enfermería traten de comprender y aplicar el modelo sobre los procesos de cambio y contribuyan a mejorar la calidad de la atención. Estos son pasos interconectados que se complementan entre sí para garantizar un cambio efectivo, por lo que, dependiendo del escenario, algunos pasos pueden ocurrir al mismo tiempo. De esta forma, el autor refuerza la importancia de no saltarse ningún paso del proceso de cambio y, cuando todos los pasos están bien dirigidos, se obtiene un resultado exitoso. Sin embargo, en el complejo mundo en el que vivimos, algunos casos no siguen estrictamente el flujo de ocho pasos, sino que el patrón de ocho pasos es el paradigma básico que se observa en los procesos de cambio exitosos^16^ ^)-(^ ^18^.

A su vez, se encontraron estudios internacionales^19^ ^)-(^ ^21^ en la Región Metropolitana de Filadelfia, en África y en el Reino Unido, que aplicaron el modelo de John Kotter en el contexto de la salud, por medio de enfermeros líderes y otros profesionales de la salud, durante la pandemia de COVID-19. Y surgieron resultados positivos del modelo de cambio de Kotter y sus conceptos de liderazgo, aunque en algunos casos los profesionales tuvieron que cambiar sus creencias, valores, comportamientos y actitudes para desarrollar continuamente nuevos conocimientos, que les permitieran a la mayoría de los profesionales adoptar un cambio exitoso^19^ ^)-(^ ^21^. Sin embargo, no se encontraron estudios en el contexto de la enfermería en América Latina.

Por consiguiente, surgió la siguiente pregunta: ¿cuáles son las percepciones sobre liderazgo de los enfermeros durante la pandemia de COVID-19, en los hospitales universitarios brasileños, según los conceptos de John Kotter? Por lo tanto, para responder esa pregunta, se presentó el siguiente objetivo: comprender las percepciones sobre liderazgo de los enfermeros durante la pandemia de COVID-19, en hospitales universitarios brasileños, a través de la concepción de los conceptos del marco de John Kotter y su modelo de cambio de ocho pasos.

## Método

### Diseño del estudio

Se trata de un estudio multicéntrico de carácter cualitativo y diseño analítico basado en los constructos teóricos de John Paul Kotter y su modelo de ocho pasos de cambio. El diseño cualitativo se consideró el más adecuado para comprender e identificar las percepciones sobre liderazgo de los enfermeros en el contexto de la pandemia. Para garantizar el rigor metodológico del estudio, se utilizaron elementos de la *checklist* para la investigación cualitativa según las directrices del *Consolidated Criteria For Reporting Qualitative Research* (COREQ)^22^.

### Lugar del estudio

El estudio se desarrolló en diez hospitales universitarios brasileños, que son campos prácticos para diferentes carreras en el contexto de la salud, incluida la enfermería. Estos atienden casos de mediana y alta complejidad para el Sistema Único de Salud (SUS)^23^, y ocho de ellos están vinculados a la Empresa Brasileña de Servicios Hospitalarios (EBSERH). Estos hospitales están ubicados en las Regiones Sur (dos hospitales, incluida la institución matriz); Sureste (dos); Noreste (dos hospitales, uno de los cuales es la institución proponente del investigador); Norte (dos); y Centro-Oeste (dos). La elección de estos diez hospitales universitarios fue, en primer lugar, intencional, a través de la interacción entre los grupos de investigación, docentes e investigadores vinculados a las instituciones educativas integradas en esos hospitales universitarios, además porque representan las cinco Regiones de Brasil y fueron designadas como instituciones de referencia para la atención de pacientes con COVID-19 por el SUS, durante la pandemia. Por lo tanto, el primer contacto con los hospitales universitarios lo realizaron profesores que eran los referentes en la coordinación del proyecto multicéntrico en cada una de las instituciones educativas.

El estudio se desarrolló en diez hospitales universitarios brasileños, que son campos prácticos para diferentes carreras en el contexto de la salud, incluida la enfermería. Estos atienden casos de mediana y alta complejidad para el Sistema Único de Salud (SUS)^23^, y ocho de ellos están vinculados a la Empresa Brasileña de Servicios Hospitalarios (EBSERH).

Estos hospitales están ubicados en las siguientes regiones: Sur (dos hospitales, incluida la institución matriz); Sureste (dos); Noreste (dos hospitales, uno de los cuales es la institución proponente del investigador); norte (dos); y Centro-Oeste (dos).

La elección de estos diez hospitales universitarios fue, en primer lugar, intencional, a través de la interacción entre los grupos de investigación, docentes e investigadores vinculados a las instituciones educativas integradas en esos hospitales universitarios, y porque representan las cinco Regiones de Brasil. Además, fueron designadas como instituciones de referencia para la atención a pacientes con COVID-19 por parte del SUS, durante la pandemia. De esta manera, el primer contacto con los hospitales universitarios lo realizaron los profesores que eran los referentes en la coordinación del proyecto multicéntrico en cada una de las instituciones educativas.

### Periodo

La recopilación de datos se realizó entre marzo de 2021 y abril de 2022.

### Población, criterios de selección, definición de la muestra

La muestra estuvo compuesta por un total de 139 entrevistados, y los participantes de la investigación eran enfermeros que trabajan en la atención de pacientes con COVID-19, y que no tenían ninguna relación laboral. Se establecieron los siguientes criterios de elegibilidad: ser enfermero y atender a pacientes con COVID-19 durante al menos tres meses, que es el tiempo que se considera necesario para tener una experiencia sobre el fenómeno abordado.

Hay que aclarar que debido a que este estudio está vinculado a un proyecto multicéntrico que utilizó métodos mixtos, en primer lugar, el proyecto más grande realizó la etapa cuantitativa, mediante muestreo por conveniencia, en la que se invitó a participar a todos los enfermeros que trabajaban en sus unidades. Y los mismos respondieron si estaban interesados en pasar a la segunda etapa, a través de entrevistas semiestructuradas.

Por lo tanto, la etapa cualitativa se realizó de forma concomitante: 1) por medio de los encuestados de la etapa cuantitativa ya agendados en el macroproyecto y que aceptaron participar en la fase cualitativa; 2) los participantes también fueron reclutados por correo electrónico, *WhatsApp* ^*®*^ institucional y por agentes de investigación presenciales (profesores de enfermería, estudiantes de grado, becarios de iniciación científica y posgrado en enfermería e instituciones vinculadas al macroproyecto, los cuales fueron entrenados y capacitados para realizar las entrevistas); 3) una vez que aceptaron participar, se les pidió a los entrevistados que recomendaran a otros enfermeros.

Los participantes que aceptaron firmaron el Formulario de Consentimiento Libre e Informado (FCLI) y participaron de las entrevistas en dos formatos: por medio de videollamadas realizadas a través de la aplicación *Google Meet* ^*®*^ y/o en un espacio reservado en su lugar de trabajo, ambas se realizaron de forma individual.

Cabe mencionar que se contó con un instrumento estandarizado para el abordaje de los participantes, que les presentaba los investigadores a los participantes, les explicaba el proyecto matriz y la duración de las entrevistas (20 a 50 min), en ese orden. 

Se realizaron reuniones *online*, mediadas por los coordinadores del proyecto matriz, para capacitar a los recolectores de datos y estandarizar los instrumentos de recolección de datos, que se basaron en el Manual de Recolección Cualitativa sobre la Gestión del Cuidado, Liderazgo de Enfermería y Ambiente de Práctica.

De esa forma, en las reuniones realizadas con la coordinación del proyecto matriz y los investigadores que forman parte de las instituciones educativas vinculadas, se determinó una fecha para iniciar las entrevistas y se realizó una prueba piloto en el HUF vinculado al proyecto matriz, que contó con la participación de un enfermero. Cabe destacar que esta entrevista no formó parte del estudio. Durante las reuniones que se llevaron a cabo con la coordinación y sus investigadores, se discutió en conjunto la saturación de datos para definir una fecha de finalización de las entrevistas.

Se incluyó la opción de una entrevista remota porque, en ese momento, la situación aún era crítica debido a la pandemia de COVID-19.

### Recolección de datos

Las entrevistas eran individuales y semiestructuradas, estaban compuestas por nueve preguntas sobre cambios, estrategias de liderazgo y gestión de los cuidados de enfermería durante la pandemia. En lo que respecta a las preguntas de caracterización de los participantes, fueron respondidas por los mismos al inicio de las entrevistas, y correspondían a: iniciales del nombre; sexo; edad; nivel de formación; tiempo de desempeño profesional; tiempo de trabajo con pacientes con COVID-19.

Los datos fueron recopilados por investigadores que formaban parte del proyecto principal en cada ubicación. El formato de la aplicación *Google Meet* ^*®*^ permitió grabar las entrevistas y cuando eran presenciales se utilizó una grabadora o un teléfono celular como grabadora de voz (mp3). Luego, las entrevistas fueron transcritas íntegramente por los investigadores de cada localidad y concluyeron cuando se alcanzaron los objetivos propuestos, acordados previamente en una reunión virtual (*Google Meet* ^*®*^ ) con todos los investigadores involucrados, en la que se definió la fecha definitiva para la finalización de todas las entrevistas en todos los hospitales.

### Análisis de los datos

Para analizar la información obtenida en las entrevistas se utilizó el método de análisis de contenido de Bardin^24^, que tiene las siguientes fases: primera, preanálisis, en la que se realizó una “lectura flotante” y exploración de contenido; segunda, exploración del material, en la que se hizo una lectura más profunda de los hallazgos, seguida de la elección de las unidades de codificación y creación de las categorías; y la tercera fase es el tratamiento de los resultados obtenidos a través de análisis e inferencia^24^.

Para ello, luego de realizar una lectura profunda de las entrevistas, se decidió: elaborar una tabla en el programa *Word* de *Microsoft Office 365*, en la que se destacaron las percepciones sobre liderazgo de los enfermeros que trabajaban durante la pandemia de COVID-19; y relacionar los hallazgos según los conceptos de Kotter y el modelo de ocho pasos de cambio. Este análisis se realizó de forma individual para cada HUF, por ende, se creó un documento *Word* para cada una de las instituciones hospitalarias. Cabe señalar que los coautores llevaron a cabo una revisión por pares, que contribuyó a la calidad de esta investigación.

También se optó por utilizar el *software webQDA* ^*®*^ , versión 3.0, para fortalecer el estudio, dado que se trata de una aplicación para investigación cualitativa que ayuda al investigador en los pasos de organización, edición y visualización de categorías y subcategorías, porque permite almacenar información de forma ordenada^25^. Para preservar el anonimato de los participantes, se los identificó con las iniciales de cada universidad a la que pertenecían, seguidas de un numeral en orden ascendente, por ejemplo: *“UFBA01; UFSC 01; UFRJ 01...”.*

### Aspectos éticos

Se cumplieron todos los preceptos éticos que involucran estudios con seres humanos, según lo determinado por la Resolución n.º 466/2012 del Consejo Nacional de Salud de Brasil^26^. Cabe señalar que el estudio fue aprobado por el Comité de Ética en Investigación (CEP) del macroproyecto, correspondiente al proyecto matriz, bajo dictamen CAAE: 38912820.3.1001.0121, y además fue aprobado por el CEP de la institución proponente de este estudio, con CAAE: 38912820.3 .2011.0049, ambos a través de la Plataforma Brasil.

## Resultados

En relación a la muestra de este estudio, fueron incluidos 139 enfermeros, que trabajaban en el cuidado de pacientes con COVID-19. La edad de los participantes osciló entre 23 años y 65 años, con una media de 32 años. Con respecto al sexo, 118 participantes eran del sexo femenino (87,41%) y 17 del sexo masculino (12,59%). En cuanto a la experiencia profesional, la mínima identificada fue de un año de servicio y la máxima de 36 años. Por último, con respecto al nivel educativo, cuatro participantes contaban con doctorado y 36 con maestría.

En el análisis temático-categórico de los datos de las entrevistas se identificaron cuatro categorías: Desarrollo de elementos esenciales para liderar el cambio: conocimiento, trabajo en equipo, comunicación; Relación de confianza y respeto; Motivación del equipo, orientándolo hacia el objetivo; y Complejidad del liderazgo y visión más amplia. Los datos que componen cada una de las mismas se presentan a continuación. 

### Desarrollo de elementos esenciales para liderar el cambio: conocimiento, trabajo en equipo, comunicación

En las declaraciones a continuación, los enfermeros destacaron la importancia del conocimiento, tanto sobre sus habilidades y capacidades que componen el cuidado de enfermería, como sobre sus capacidades para liderar equipos. Según ellos, este conocimiento permite que todos se preparen para realizar adecuadamente acciones a fin de lograr los objetivos comunes. Por ende, esta categoría se relaciona con algunos elementos clave del modelo de Kotter, como el primer paso, sentido de urgencia, en la que el conocimiento de las competencias y habilidades necesarias para el cuidado de enfermería y el liderazgo de equipos es fundamental para crear conciencia sobre la necesidad de cambio; el segundo paso, coalición, es importante para reunir a personas con diferentes conjuntos de habilidades y conocimientos para liderar el cambio de manera efectiva; el cuarto paso, comunicar la visión, debe garantizar que todos comprendan los objetivos del cambio y estén de acuerdo con ellos.

*Liderar es un proceso en el que se aprende a desarrollar habilidades* [...] *Hay que tener humildad para aprender de las personas* [...] *y la comunicación es uno de los pilares del liderazgo. Un líder que no comunica no tiene un buen liderazgo, porque no se lidera solo. Si lideras solo, no eres un líder, eres un jefe.* (UFBA 01)

[...] *mi condición como líder es participar siempre con el equipo, estar siempre con ellos, y no es sólo motivacional, no sólo educativo; hay que intercambiar conocimientos, porque nadie lo sabe todo. Es una adquisición de conocimiento, es un intercambio, y muchos de mis compañeros que son técnicos forman parte de mi equipo* [...]. (UFSC 09)

*Hay tres cosas esenciales para que el liderazgo sea eficiente: el conocimiento, la actitud y la otra es lo que haces con tu trabajo con tu equipo. La forma en que te comportas con el equipo es lo que te convierte en un líder. Entonces, ante la pandemia, el punto que permitió llevar a cabo una buena atención fue el conocimiento. Para convertirse en líder es necesario tener conocimiento.* (UFAM 09)

*El enfermero debe tener conocimiento, conocimiento de sus acciones, conocimiento de sus competencias, de la competencia específica del equipo de enfermería, porque hay que saber delegar. Entonces, si no tiene este conocimiento, no puede delegar las acciones importantes para brindar atención. También debe conocer las rutinas, los protocolos de seguridad del paciente, porque si no los sabe, no podrá ver esto en la práctica* [...] *debe tener actitud, esas tres características: conocimiento, habilidad y actitud* [...]. (UFRN 03)

*No conozco ningún enfermero que no sea líder. No sé, si estás en la práctica clínica y estás ahí, tienes que gestionar ese equipo* [...] *Tienes que conocer cada vez más a tu equipo, tener cada vez más conocimiento científico, algunos enfermeros olvidan que ese conocimiento es importante* [...]. (UNIFESP 02)

*No tengo ningún problema en hablar con la gente, en comunicarme. Esto le hace muy bien al paciente, porque nos comunicamos* [...]. *En mi equipo, veo esto, que es un problema que existía y que hoy ya no existe, el tema de la comunicación, y también le doy la libertad a los técnicos de acudir a mí y hablar* [...] *esa gran articulación, creo que es muy buena* [...]. (UFRJ 10)

### Relación de confianza y respeto

En esta segunda categoría surgió nuevamente la importancia del trabajo en equipo, pero ahora basado en una relación de confianza y respeto. Según los participantes, sin estos elementos es difícil que un líder pueda ejercer el liderazgo. Cabe destacar también que en los discursos se menciona la empatía, especialmente ante el escenario de la pandemia, en el que el respeto y la colaboración fueron fundamentales. De esa forma, es posible relacionar los hallazgos con los preceptos de Kotter y su modelo, debido a que el autor destaca los elementos de confianza y respeto mutuos. Por ejemplo, en el segundo paso que es la coalición, es necesario que haya confianza y respeto entre el líder y su equipo para trabajar de manera más colaborativa y efectiva para lograr los objetivos comunes del cambio.

*En lo que respecta a las características de un líder: lo primero es confiar en el equipo que tiene; tener empatía; ponerse la camiseta del equipo; trabajar con el equipo para ayudar y estar en la primera línea con los demás* [...] *ser el primero en poner el pie en una línea de combate y el último en quitarlo* [...] *tienen que estar con ellos en la batalla , de la mano, o al frente, o cuidando la retaguardia detrás, tener mucha inteligencia emocional* [...] *porque, si el líder se desespera, se acabó, se acabó el equipo, se acabó todo, se acabó la confianza; tener confianza en el técnico y transmitirle confianza también. Sabes, estás ahí para acompañarlo.* (UFMT 04)

*Con respecto a los atributos de personalidad, creo que tiene que tener empatía, empatía para saber ponerse en el lugar del otro, entender hasta qué punto el equipo necesita apoyo, si tiene dificultades, cómo va a poder ayudar al equipo* [...]. (UFRN 03)

*El líder es el reflejo de su equipo, si trabajas con tu técnico, con tu compañero, le vas a transmitir seguridad. Tienes que ser bueno en lo que haces y tienes que respetar a tus compañeros, independientemente de la carrera que tengan.* (UFMS 02)

*Creo que es una relación más horizontal que vertical, sin dejar de respetar las jerarquías, pero el liderazgo, para que se entienda y se comprenda, debe escuchar al liderado, porque ahí es donde se establece una relación de confianza y respeto* [...]*.* (UFBA 07)

[...] *Voy a transmitirle confianza a mi equipo que puede contar conmigo. Entonces, esto lo sentimos mucho en cuando enfrentábamos el COVID-19, porque muchas personas, muchos compañeros, llegaban en muy malas condiciones, ya sea porque se enfermó algún familiar, o porque no pudieron ver a su hijo, porque tenían que mantener distancia, no podían abrazarse, no podían besarse* [...]. (UFSC 09)

### Motivación del equipo, orientándolo hacia el objetivo

En esta tercera categoría, se presentan las percepciones de los enfermeros sobre los atributos para motivar, inspirar e influir en las personas. Para ellos, un líder debe tener el coraje de liderar, delegar sabiamente y ejercer ese papel. De esa forma, podemos relacionarla con los conceptos de Kotter y su modelo, destacando el primer paso sentido de urgencia y el tercer paso comunicación de la visión. Debido a la importancia que tiene establecer un sentido de urgencia para motivar a las personas a tomar medidas para lograr los objetivos del cambio, lo cual fue fundamental durante la pandemia de COVID-19. Un líder motivado e inspirador puede comunicar de forma clara y convincente los objetivos del cambio y el propósito detrás del mismo.

[...] *influir satisfactoriamente en las personas; el líder tiene que tener habilidades, cualidades para motivar a su equipo* [...] *y presentar los objetivos, cuáles son los objetivos de estar ahí en ese sector, en esa organización, qué podemos hacer cada día mejor* [...] *realmente motivar a la gente, a los empleados, que somos un equipo, que no trabajamos solos, yo como enfermera no puedo trabajar sola, tengo un técnico de enfermería, hay un médico, hay un psicólogo, hay un fisioterapeuta, están todos, es un equipo.*[...]. (UFMT 01)

[...] *liderazgo surge de influir en las personas, inspirarlas y delegar sabiamente; está para indicar la dirección para un objetivo. Yo creo que ser líder no es tener un puesto, la gente va a ver, digámoslo así, la gente lo va a verlo como líder no por un puesto, sino por sus actitudes, la postura y cómo se comporta* [...]. (UFPA 02)

*Creo que el coraje, el coraje es un aspecto que hay que tener y mucho. Coraje para ser líder, coraje para llamar a quien es necesario para hablar, cuando sea necesario, a veces hasta para enfrentar a los pacientes, creo que se necesita coraje.* (UNIFESP 04)

### Complejidad del liderazgo y visión más amplia

Las afirmaciones de esta última categoría revelan la complejidad y dificultad de liderar, que, para los enfermeros durante la pandemia de COVID-19, resultó ser aún más desafiante, ya que los obstáculos eran grandes y, muchas veces, se concentraban en las unidades de cuidados intensivos, una de las más críticas en un hospital. Por ende, el enfermero debía tener una visión más amplia, ir más allá de la teoría y experimentar y adaptarse a la práctica día a día. Podemos relacionar que Kotter señala que una visión más amplia es fundamental para comprender y lidiar con los complejos desafíos que enfrentan los líderes ante una crisis, pero que la parte difícil es cambiar el comportamiento de las personas.

*El liderazgo es muy difícil, el tema de la aceptación, tanto del servicio como de seguir normas y rutinas, presenta mucha resistencia. Es la resistencia a cualquier cambio. Entonces, tenemos que adaptarnos, encontrar maneras de convocarlos para que podamos continuar. Tiene que ser como un intercambio. No soy líder sola, tenemos que intercambiar experiencias y trabajar juntos, no tiene sentido querer imponer nada. [...] El liderazgo en Enfermería es un desafío enorme. (UFPA 07)*

*El liderazgo es un rol muy complejo, creo que sobre todo en una unidad de cuidados intensivos, ya que el rol del enfermero es, en mi opinión, el rol más importante dentro de la UCI hoy en día, porque somos una mezcla de todo lo que sucede. Creo que ser líder va mucho más allá del concepto que sacamos de la literatura, creo que es una experiencia que tiene que venir de un contexto ético muy positivo que es solo la experiencia en sí, desde una perspectiva más humanizada [...]. (UFMT 02)*

*Creo que el liderazgo va más allá de liderar el sector, de ver las cuestiones técnicas, la logística, la estructura. Creo que tiene que haber una visión holística de punta a punta del sector, de cada rincón, de cada paciente, de cada cosa, creo que el líder tiene que desarrollar esa visión. Con respecto al paciente, no sólo con respecto al cuidado, brindar atención y observar su individualidad, la cuestión psicológica, pensar en la familia [...]. (UFRJ 05)*

*Creo que es un atributo, pongámoslo así, que debemos tener, en el sentido no sólo de resolver los conflictos del equipo, sino de ver de forma más amplia cómo eso afecta la atención y qué puedo hacer yo o qué puede hacer mi equipo para mejorar la atención, cuidando al mismo tiempo a los pacientes y al personal y cuidando los procesos de trabajo. Entonces, creo que es en ese sentido poder ver de forma amplia todo lo que puede hacer el enfermero para mejorar este proceso, tanto para los pacientes, como para el equipo y para la institución. (UFSM 04)*

## Discusión

En el contexto de la pandemia de COVID-19, se produjeron cambios constantes en las prácticas de atención de salud, y el papel de los líderes de enfermería, que estuvieron en la primera línea, se destacó. En este sentido, los discursos permitieron comprender las percepciones de esos profesionales sobre el liderazgo que ejercen, y discutir las concepciones de los conceptos del marco de John Kotter y su modelo de cambio de ocho pasos.

En primer lugar, el estudio aborda elementos esenciales del liderazgo, como el conocimiento, el trabajo en equipo y la comunicación. Surgió una comunicación bidireccional y sensible al otro, debido a la pandemia, que exigía una respuesta diligente y atenta al sentido de urgencia de la situación. Se destacó el liderazgo basado en el conocimiento y el diálogo respetuoso entre el enfermero y su equipo, que contribuyó a articular el trabajo en equipo.

Por ende, según el punto de vista de Kotter, los líderes deben reunir ciertas capacidades, como acumular conocimiento relevante que contribuya a una visión más amplia. También es necesario desarrollar habilidades de liderazgo relacionadas con la visión, la comunicación y la motivación para todas las etapas de un proceso de cambio^17^ ^),(^ ^27^.

En Taiwán, el modelo de ocho pasos de Kotter se utilizó en una Red Internacional de Hospitales y Servicios de Salud para certificar el Hospital Amigo de las Personas Mayores, y destacó que el conocimiento y las actitudes de los empleados son fundamentales para lograr un cambio exitoso. Además, el modelo contribuyó a la adhesión de los empleados centrándose en el éxito, y fue importante, durante todas los pasos del proceso, adoptar una comunicación clara para que todos los involucrados pudieran comprender^28^.

En Brasil vemos resultados similares y se destaca la importancia del conocimiento de los enfermeros sobre el COVID-19, los aspectos, las formas de transmisión y la manera de combatirlo. Se identificó que los principales medios de aprendizaje que utilizaron los enfermeros para enfrentar la enfermedad se centraron en buscar información por su cuenta, en Internet o la televisión, y en participar en capacitaciones promovidas por las respectivas instituciones de salud a través de la educación continua. Sin embargo, según el estudio, la base del aprendizaje estaba en las experiencias de los sujetos involucrados^29^.

Además, en Brasil, se dio una situación similar, en la que se destaca que los enfermeros y otros profesionales de la salud, que se desempeñaban como gestores, debían basarse estrictamente en la evidencia científica y las recomendaciones de los organismos internacionales y nacionales competentes para tomar decisiones y llevar a cabo acciones urgentes sobre el COVID-19. Fue evidente el protagonismo de los enfermeros en la conducta adoptada^30^.

Por ende, es evidente hasta que punto el enfermero líder debe actualizar su conocimiento, tanto dentro como fuera de su contexto laboral, y que debe ampliar su visión. La actitud de estar siempre actualizado le permitirá incluso sentirse preparado para liderar a su equipo y, por lo tanto, ser capaz de garantizar la calidad de la atención.

El estudio destacó la confianza y el respeto que había entre el enfermero líder y su equipo, lo que también se relaciona con los preceptos de Kotter. Según el autor^16^ ^)-(^ ^17^, durante el ejercicio del liderazgo del cambio, los buenos equipos aprenden a operar con confianza y compromiso emocional, mientras que, en el liderazgo menos inspirador, confían sólo en una sola persona o en nadie^16^ ^)-(^ ^17^.

Kotter incluso considera que es extremadamente difícil llevar a cabo transformaciones importantes, hecho que, según él, requiere un grupo de trabajo fuerte y valora precisamente la etapa de coalición administrativa (creación de una comisión administrativa fuerte). Para él debe haber confianza y respeto, de lo contrario, una persona que genera falta de confianza puede destruir el trabajo de todo un equipo^16^ ^)-(^ ^17^ ^),(^ ^27^. 

Por lo tanto, un estudio^31^ que abarcó la motivación, la inspiración y el coraje para asumir este rol de líder, concluyó que, en tiempos tan inciertos como la pandemia de COVID-19, los líderes deben tener resiliencia para enfrentar las adversidades y aprender de ellas. Para eso, deben preparar a la institución para aprender y evitar una crisis de liderazgo, demostrando credibilidad, coraje y compasión. También es necesario que les enseñen a sus equipos valores esenciales, como empatía, confianza, transparencia e integridad, para comprender la necesidad del cambio. Por ende, le corresponde al líder tener el coraje de decir la verdad y liderar en medio de la incertidumbre^31^.

En Estados Unidos (EE.UU.), una investigación se enfocó en una realidad diferente y advirtió sobre la gravedad de los escenarios en los que no hay confianza en los líderes. Los informes destacaron situaciones en las que los profesionales de la salud demostraron dolor, frustración e ira y necesitaban el apoyo de sus líderes y no lo tuvieron. Además, de la falta de espacios seguros para el diálogo abierto, de la falta de comunicación transparente y la impotencia al atender al paciente, que están fuera de discusión. La suma de estos atributos tuvo un impacto significativo en el ámbito de trabajo de dichos profesionales y contribuyó a la ruptura de la relación de confianza e incluso afectó la capacidad de confiar en sí mismos^32^.

Por eso, el concepto de Kotter enfatiza que, si no hay confianza dentro del equipo, todo el proceso de cambio no fluirá^16^ ^)-(^ ^18^. Por lo tanto, el enfermero líder debe estar atento y permanecer fiel al objetivo de promover el trabajo en equipo de manera armoniosa, para beneficio de todos los involucrados.

Por último, las declaraciones destacaron la dificultad y complejidad del liderazgo, y lo difícil que es ejercerlo, especialmente en el campo de la enfermería y en el contexto de la lucha contra el COVID-19. Ser líder, en estas situaciones, puede ser duro.

Liderar el cambio es una tarea importante y difícil para los líderes de la atención en salud, por lo que muchas personas, organizaciones o comunidades prefieren permanecer en sus “zonas de confort”, lo que implica un problema para el cambio. Otro obstáculo es lograr que la gente cambie su actitud^28^. Kotter destaca que el gran desafío del líder es cambiar el comportamiento de las personas, y que requiere un constante “ver-sentir-cambiar”^17^ ^),(^ ^27^. Sin embargo, está claro que la pandemia de COVID-19 generó en los enfermeros líderes la sensación de que necesitaban adaptarse a los tres pilares mencionados.

Una investigación muestra que esta pandemia planteó desafíos para el liderazgo del enfermero, pero que ese profesional se propuso lograr que el equipo evolucione y valorar a los demás, basándose en la confianza y la resiliencia del equipo^33^. Una investigación reciente, que arrojó resultados similares, destacó el coraje, el compromiso y la autorrealización profesional que demostraron los trabajadores de la salud para enfrentar el COVID-19. Sin embargo, revela que, en el trabajo de los profesionales de enfermería, el sufrimiento aumentó significativamente debido a que a un escenario que cambiaba constantemente se le sumaron altas exigencias, además destaca la penuria y el aumento del volumen de trabajo debido a la complejidad del cuidado de los pacientes^34^.

Por lo tanto, el estudio contribuye a ayudar y fortalecer el liderazgo de los enfermeros en entornos de constante cambio e incertidumbre, como el de la salud; a través de los constructos de John Kotter y su modelo de cambio, dado que es una herramienta de liderazgo muy importante para apoyar a los líderes en los procesos de cambio de las organizaciones^16^, que hace hinca pie en la importancia de involucrar a las personas para que el proceso ocurra de manera efectiva. Se espera que, ante una crisis o escenario inesperado como el de la pandemia, los enfermeros líderes sean capaces de reflexionar y fortalecer el trabajo en equipo, comunicando con claridad los cambios necesarios en las prácticas de atención a la salud y mejorando la asistencia sanitaria.

En cuanto a las limitaciones del estudio, el período de recolección de datos se produjo en momentos aún complejos en términos de infección y mortalidad por COVID-19. Si bien la estrategia de vacunación ya había comenzado, era un momento delicado, en el que, en general, los profesionales estaban sobrecargados de trabajo. Por ello, aunque las entrevistas habían sido programadas previamente, hubo casos en los que fue necesario reprogramar y otros en los que los participantes abandonaron el estudio. Por otro lado, esta investigación permite que se lleven a cabo discusiones sobre la concepción de liderazgo en el campo de enfermería, y sus resultados contribuyen a cualificar la práctica profesional del enfermero líder.

## Conclusión

Se concluye que las percepciones sobre liderazgo de los enfermeros brasileños que trabajaban en hospitales universitarios, durante la pandemia de COVID-19, presentan características consistentes con los preceptos de John Kotter. Esto suscita reflexiones para que los enfermeros líderes emprendan cambios en la práctica, e incluso para otros profesionales de la salud que ocupan posiciones de liderazgo.

Cabe destacar que, en las percepciones de los enfermeros se puede ver la presencia de elementos esenciales para el liderazgo que, a pesar de ser desafiantes, son sumamente necesarios y fortalecedores para que todo el proceso de cambio ocurra con calidad y eficacia. Algunos de estos elementos para liderar cambios hacen referencia a la búsqueda de adquirir múltiples conocimientos, que implica el intercambio de conocimiento entre el enfermero líder, su equipo de enfermería y otros profesionales de la salud. Por ende, hay que partir de un liderazgo basado en la comunicación respetuosa, que facilite el trabajo en equipo a través de una relación de confianza y respeto. Los enfermeros reconocieron lo complejo que es liderar, que es arduo y difícil, especialmente en el contexto de la pandemia de COVID-19, y que se vuelve más complicado aún por la resistencia al cambio.

Cabe destacar que es importante que se realicen nuevas investigaciones que aborden el tema del liderazgo en enfermería, tanto por su complejidad como para actualizar el conocimiento y hacerlo más comprensible para implementarlo/aplicarlo en la práctica. Se considera que es cada vez más necesario desarrollar esta competencia en escenarios de cambio y de grandes impactos.
